# Copolymerization and terpolymerization of carbon dioxide/propylene oxide/phthalic anhydride using a (salen)Co(III) complex tethering four quaternary ammonium salts

**DOI:** 10.3762/bjoc.10.187

**Published:** 2014-08-05

**Authors:** Jong Yeob Jeon, Seong Chan Eo, Jobi Kodiyan Varghese, Bun Yeoul Lee

**Affiliations:** 1 Department of Molecular Science and Technology, Ajou University, Suwon 443-749 Korea

**Keywords:** carbon dioxide, CO_2_ chemistry, cobalt complex, phthalic anhydride, propylene oxide, terpolymerization

## Abstract

The (salen)Co(III) complex **1** tethering four quaternary ammonium salts, which is a highly active catalyst in CO_2_/epoxide copolymerizations, shows high activity for propylene oxide/phthalic anhydride (PO/PA) copolymerizations and PO/CO_2_/PA terpolymerizations. In the PO/PA copolymerizations, full conversion of PA was achieved within 5 h, and strictly alternating copolymers of poly(1,2-propylene phthalate)s were afforded without any formation of ether linkages. In the PO/CO_2_/PA terpolymerizations, full conversion of PA was also achieved within 4 h. The resulting polymers were gradient poly(1,2-propylene carbonate-*co*-phthalate)s because of the drift in the PA concentration during the terpolymerization. Both polymerizations showed immortal polymerization character; therefore, the molecular weights were determined by the activity (g/mol-**1**) and the number of chain-growing sites per **1** [anions in **1** (5) + water (present as impurity) + ethanol (deliberately fed)], and the molecular weight distributions were narrow (*M*_w_/*M*_n_, 1.05–1.5). Because of the extremely high activity of **1**, high-molecular-weight polymers were generated (*M*_n_ up to 170,000 and 350,000 for the PO/PA copolymerization and PO/CO_2_/PA terpolymerization, respectively). The terpolymers bearing a substantial number of PA units (*f*_PA_, 0.23) showed a higher glass-transition temperature (48 °C) than the CO_2_/PO alternating copolymer (40 °C).

## Introduction

**Scheme 1:**
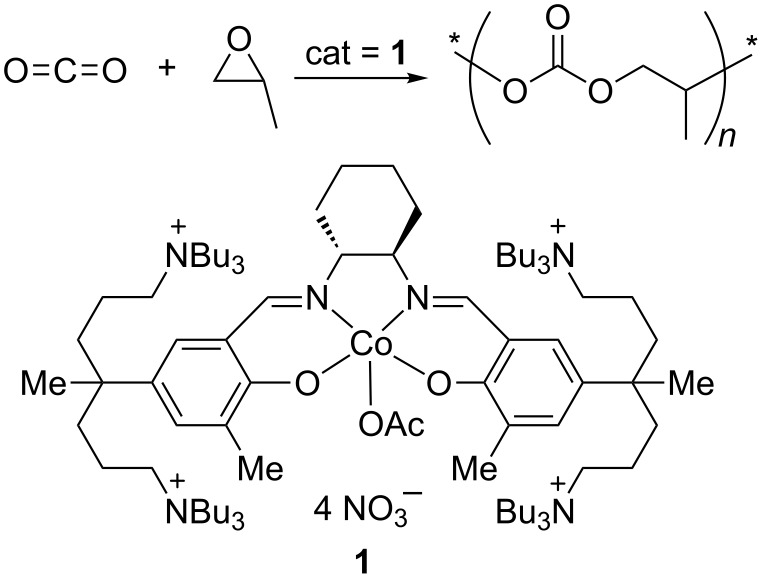
Synthesis of poly(propylene carbonate) (PPC) using catalyst **1**.

Carbon dioxide (CO_2_) can be utilized to prepare aliphatic polycarbonates through coupling reactions with epoxides [1–6]. The pioneering work for this copolymerization was introduced by Inoue in 1969 [7]. Eventually, a highly active and efficient catalyst was developed based on the concept of combining (salen)Co(III) units with quaternary ammonium salts in a molecule [8–12]. The highly efficient catalyst (**1** in Scheme 1) showed a high turnover frequency (TOF, 16,000 h^−1^), high molecular weight (*M*_n_, up to 300,000), and good selectivity (>99%). These promising performances motivated to construct a continuous-process pilot plant in industry [13]. Currently, many hurdles in the preparation of **1** on a large scale have been overcome, and an economical synthesis on the 100 kg scale has been achieved [14–16], along with precise control of the molecular weight and chain topology, facilitating the applications of these attractive materials [14,17–19]. Now, the production of poly(propylene carbonate) (PPC) using catalyst **1** is at the stage of the final decision regarding a commercial investment. Another success story using double metal cyanide (DMC) catalysts has been reported recently [20]. The DMC catalysts provided low-molecular-weight CO_2_/PO copolymers containing significant numbers of ether linkages [21–22].

PO and ethylene oxide (EO) are bulk chemicals produced annually on the million-ton scale, and the focus in industry has been on CO_2_/PO and CO_2_/EO copolymerizations. Catalyst **1** is also highly active in CO_2_/EO copolymerizations [23]. Even though the CO_2_/PO or CO_2_/EO copolymer itself shows some advantageous properties such as biodegradability, good adhesiveness, good barrier properties, and clean burning properties, the incorporation of a third monomer has also been pursued to improve the properties and hence facilitate its use in more widespread applications. For example, the terpolymerization of CO_2_/PO/cyclohexene oxide (CHO) was successful, providing resins, of which the glass-transition temperatures (*T*_g_) were modulated in the range of 50–100 °C according to the mole fraction of the incorporated CHO [24–25]. However, the feeding of a third monomer such as CHO gives rise to intrinsic problems in terms of commercial operation. The third monomer is not completely consumed, and the remaining CHO should be recovered and recycled, which is a severe burden because of its high boiling point (130 °C). Moreover, the toxic CHO should be removed completely from the resin for use in our daily life. In this work, we demonstrate the complete incorporation of the third monomer of phthalic anhydride (PA) in CO_2_/PO copolymerizations using catalyst **1**.

## Results and Discussion

### PO/PA copolymerizations

Alternating copolymerizations of epoxides and cyclic anhydrides using a diiminate zinc catalyst as well as a chromium(III) salen complex have been reported [26–27]. Long reaction times (>10 h) were needed to reach full conversion, and the average molecular weights were in the region of several ten thousand. When a zinc glutarate catalyst was used in PO/PA copolymerization, significant numbers of ether linkages were generated through consecutive PO incorporation [28].

**Scheme 2:**
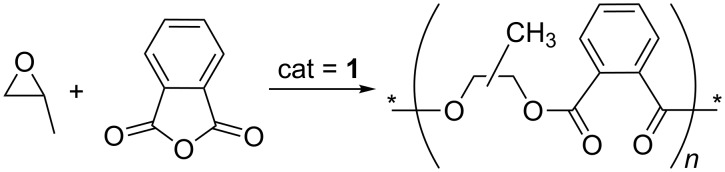
PO/PA alternating copolymerization.

**Figure 1:**
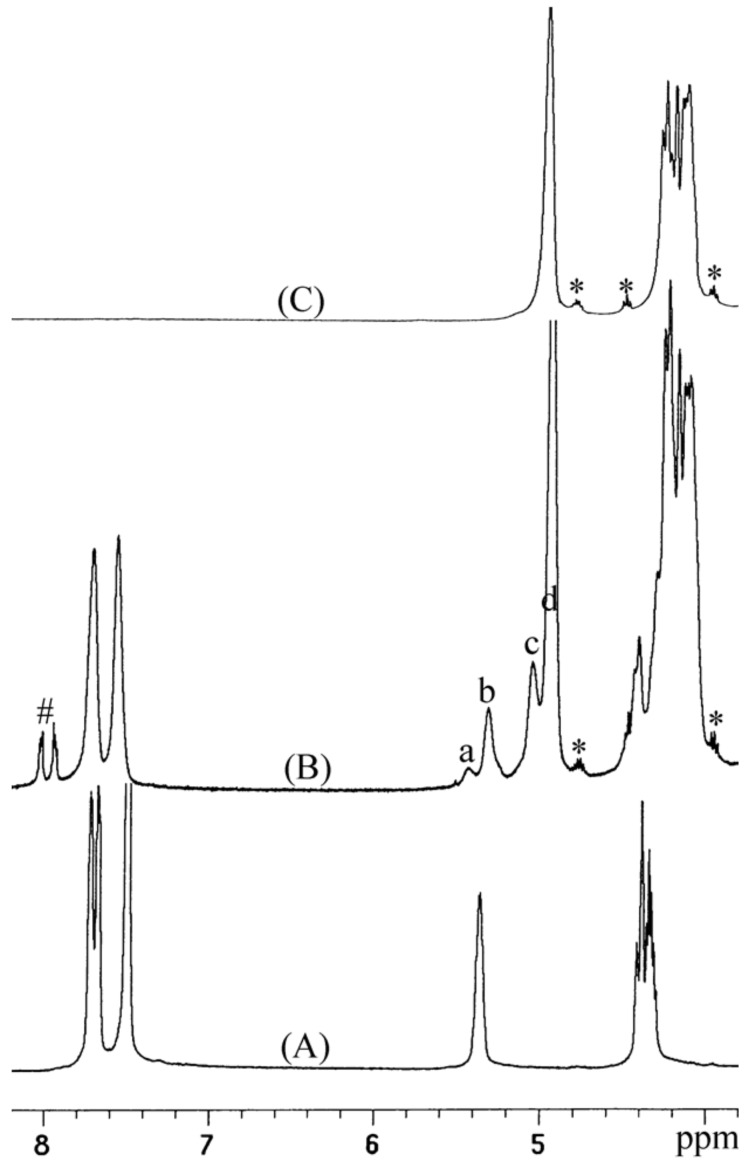
^1^H NMR spectrum of crude products in the PO/PA alternating polymerization (A, entry 1 in Table 1), PO/CO_2_/PA terpolymerization (B, entry 3 in Table 2), and CO_2_/PO alternating copolymerization (C) ("a" signal for ester-C*H*(Me)CH_2_-ester; "b" signal for ester-C*H*(Me)CH_2_-carbonate; "c" signal for ester-CH_2_C*H*(Me)-carbonate; "d" signal for carbonate-CH_2_C*H*(Me)-carbonate; "*" signals for propylene carbonate; "#" signals for unreacted PA).

When PA (1.00 g) and PO (10.4 g) were reacted using catalyst **1** (3.0 mg; **1**/PA/PO = 1:3,750:100,000) at 80 °C for 3 h, 100% conversion of PA was achieved (entry 1 in Table 1). The ^1^H NMR spectrum indicated that no PA remained in the resulting solution, and that the generated polymer was strictly alternating with no ether linkages (Scheme 2, Figure 1(A)). The isolated polymer mass was 1.39 g, in exact agreement with that calculated (1.39 g) on the basis of full conversion of 1.0 g PA to the strictly alternating PO/PA copolymer. Three aromatic signals were observed in the ^1^H NMR spectra at 7.71, 7.68, and 7.50 ppm in 1:1:2 ratios (Figure 1(A)). The OC*H*(Me) signal was observed at 5.35 ppm, while the C*H*_2_O signal was observed at 4.3–4.5 ppm. When a larger amount of PA (2.0 g) was added, the conversion of PA was 76% after 3 h (Table 1, entry 2) and 96% after 4.5 h (Table 1, entry 3), but full conversion of PA was achieved after running the copolymerization for 5.0 h (Table 1, entry 4). When 3.0 g PA was added, the conversion of PA was very low (16%), and after polymerization, a lot of unreacted PA was deposited as a solid. The solubility of PA in PO was limited; 3.0 g of PA might not dissolve fully in 10 g of PO even at a high temperature of 80 °C. Catalyst residues were removed completely through filtration with a short pad of silica gel after the polymerization. After filtration, the light orange solution became colorless, and the isolated polymer was also colorless (see Supporting Information File 1). If PA was not fully converted, the polymers were obtained as admixtures with unreacted PA (Table 1, entries 2 and 3).

**Table 1:** PO/PA alternating copolymerization results with catalyst **1**.^a^

Entry	PA (g)	EtOH (mg)	Time (h)*^b^*	PA conversion (%)	Yield^c^ (g)	Activity (Kg/g-cat)	*M*_n_^d^	*M*_w_/*M_n_*^d^	*T*_g_^e^ (°C)

1	1.0	0	3.0	100	1.39 (1.39)	0.46	59,000	1.61	65
2	2.0	0	3.0	76	(2.2)	(0.73)	80,000	1.26	62
3	2.0	0	4.5	96	(2.7)	(0.90)	116,000	1.27	65
4	2.0	0	5.0	100	2.70 (2.78)	0.90	167,000	1.21	65
5	2.0	5.0	5.0	100	2.70 (2.78)	0.90	17,000	1.43	63
6	2.0	10	5.0	100	2.63 (2.78)	0.88	11,000	1.42	60
7	2.0	15	5.0	100	2.54 (2.78)	0.84	9,000	1.40	58
8	2.0	20	5.0	100	2.48 (2.78)	0.83	6,000	1.40	55

^a^Polymerization conditions: PO (10.4 g, 180 mmol), catalyst **1** (3.0 mg, 1.8 μmol), temperature (80 °C). ^b^Including heating time of ca. 50 min. ^c^Values in parentheses calculated from the conversion. ^d^Determined on GPC using a polystyrene standard. ^e^Determined on DSC.

**Figure 2:**
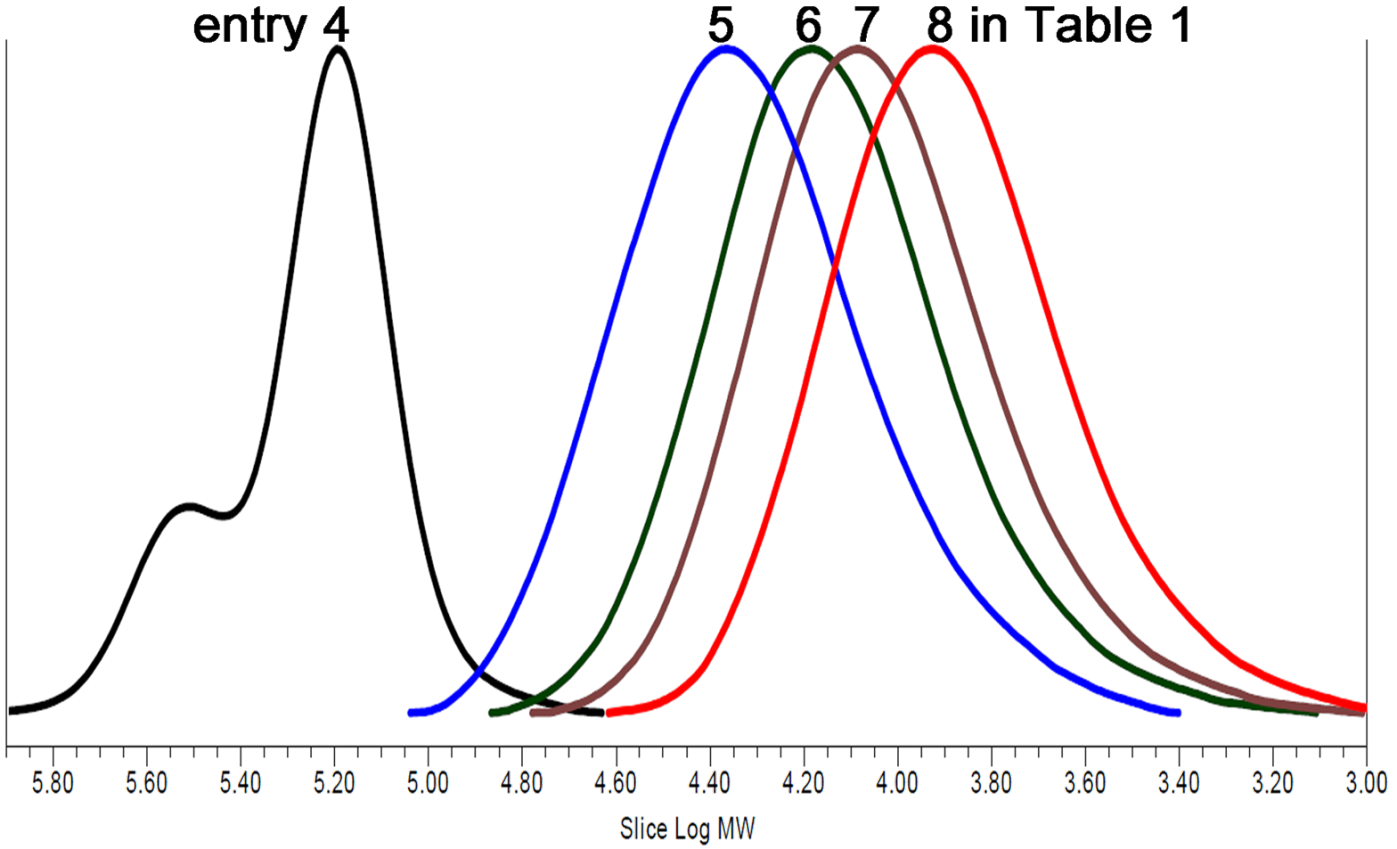
GPC curves of the PO/PA copolymers.

At the full conversion of 1.0 g PA, the number average molecular weight (*M*_n_) of the resulting polymer was 59,000 (Table 1, entry 1). Upon increasing the PA feed amount to 2.0 g and achieving full conversion, a high-molecular-weight polymer with *M*_n_ = 167,000 was obtained (Table 1, entry 4). As the reaction time (and consequently, the PA conversion) increased, the number average molecular weight (*M*_n_) increased gradually with a narrow molecular weight distribution (*M*_w_/*M*_n_ ca. 1.2) preserved in all cases, indicating living or immortal polymerization (Table 1, entries 2–4). Bimodal distributions were observed in the GPC curves (Figure 2). The peak molecular weight in the high-molecular-weight mode was always twice that in the low-molecular-weight mode. The chains in the high-molecular-weight mode were attributed to those grown biaxially from water, which was present as an impurity, while those in the low-molecular-weight mode were grown from the nitrate and acetate anions in **1**. The numbers of polymer chains generated per **1**, which was calculated from the yield (g) and *M*_n_ values [yield/(*M*_n_ × (mole of **1**)], were 13, 15, 13, and 9 for the samples in Table 1, entries 1–4, respectively. These numbers were roughly in agreement with the sum of the number of anions in **1** (5) and the number of water molecules per **1** present as an impurity (8, 10, 8, and 4, respectively). The portion of the high-molecular-weight mode (that is, the portion of chains grown from water molecules) relative to the portion of the low-molecular-weight mode decreased in the GPC curves in the order of entries 2, 3, and 4 (see Supporting Information File 1). This order was in accord with the number of water molecules calculated above from the yield and *M*_n_ values (10, 8, and 4, respectively). These observations indicated that the polymer chains grew uniformly from the anions in **1** and water molecules with immortal polymerization character. Water molecules might be incorporated into the polymerization system from various sources such as PO, CO_2_ gas, catalyst, and the reactor surface. The amount fluctuated batch by batch in the lab scale polymerization.

Upon the addition of ethanol, which was expected to act as a chain-transfer agent, the *M*_n_ values decreased significantly and were regulated precisely by the amount of ethanol fed (Table 1, entries 5–8). With 5.0 mg of ethanol fed at the PA feeding of 2.0 g, full PA conversion was achieved by running for 5 h, and the *M*_n_ value of the resulting polymer was 17,000, indicating that the ethanol worked well as a chain-transfer agent (Table 1, entry 5). As the amount of ethanol was increased to 10, 15, and 20 mg, full conversions were also achieved in 5 h, and the *M*_n_ values decreased systematically to 11,000, 9,000, and 6,000, respectively. The number of polymer chains per **1**, calculated from the yield and *M*_n_ values [yield/(*M*_n_ × (mole of **1**)], were 92, 141, 173, and 259 for the samples obtained with 5, 10, 15, and 20 mg ethanol feeding, respectively. These numbers were in rough agreement with the sum of the number of ethanol molecules per **1** (60, 120, 180, and 240, respectively) and the number of anions in **1** (5). The polymer chains were grown uniformly from the fed ethanol and the anions in **1** with immortal polymerization character. The portion of the chains grown biaxially from impurity water molecules was relatively small, and unimodal distributions were observed when ethanol was fed. The *T*_g_ value of the PO/PA copolymer was 65 °C when the molecular weight was high (mostly *M*_n_ > 80000, Table 1, entries 1–4), and decreased gradually from 63 °C to 55 °C as *M*_n_ was lowered from 17,000 to 6,000 (Table 1, entries 5–8).

### PO/CO_2_/PA terpolymerizations

The terpolymerization of CHO/CO_2_/diglycolic anhydride using a diiminate zinc catalyst has been reported. Here, the block copolymer of poly(ester-*block*-carbonate) was formed through the faster reaction of CHO/anhydride coupling, and after complete conversion of the anhydride, the carbonate block was grown by CO_2_/CHO coupling [29]. Porphyrin Al or Cr(III) complexes also generated block copolymers in PO/CO_2_/PA terpolymerizations [30]. The terpolymerizations of CHO/CO_2_/PA and PO/CO_2_/maleic anhydride using a zinc glutarate catalyst have also been reported, where random copolymers containing some ether linkages were generated [28,31].

**Scheme 3:**
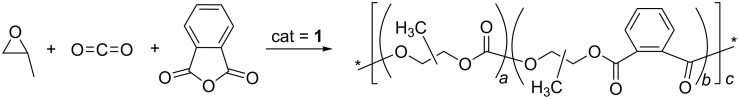
CO_2_/PO/PA terpolymerization.

When CO_2_ gas was also pressurized under the PO/PA copolymerization conditions [PA (1.00 g), PO (10.4 g), **1** (3.0 mg; **1**/PA/PO = 1:3,750:100,000), 80 °C, 3 h], all the fed PA was consumed (entry 4, Table 2). No signals due to unreacted PA were observed in the ^1^H NMR spectrum of the resulting crude product dissolved in THF-*d*_8_, in which both PA and the resulting polymer were freely soluble. Upon shortening the reaction time to 1.5, 2.0, and 2.5 h, unreacted PA signals were observed at 8.0 and 7.9 ppm along with the resulting polymer signals (Figure 1(B)). In all cases, negligible amounts of propylene carbonate were generated (less than 2 mol % per consumed PO) and no ether linkages were formed (Scheme 3). The conversions of PA were calculated simply from the ^1^H NMR spectra by using the formula (*I*_7.5-7.8_)/(*I*_7.5-7.8_ + *I*_7.9-8.1_), where *I* is the integrated value of the region defined by the subscript. As the reaction time increased, the PA conversion increased (Table 2, entries 1–4), and full conversion of PA was achieved with the formation of 6.5 g of polymer at a reaction time of 3.0 h (including the heating time of ca. 50 min). The formation of 6.5 g of polymer corresponded to a commercially acceptable high activity of 2.2 kg-polymer/g-catalyst. The CO_2_ pressure decreased monotonously from 35 to 32 bar up to the cutoff time of 3 h, indicating that the catalyst was not deactivated during the polymerization. The turnover number (TON = mole of consumed PO/mole of **1**) and turnover frequency (TOF) at the full PA conversion were calculated to be 32,000 and 12,000 h^−1^, so the performance of **1** was not deteriorated by feeding PA. In the absence of PA (that is, in CO_2_/PO copolymerizations), catalyst **1** showed a TOF of approximately 16,000 h^−1^.

**Table 2:** CO_2_/PO/PA terpolymerization results with catalyst **1**.^a^

Entry	PA (g)	EtOH (mg)	Time (h)^b^	PA Conversion (%)	*f*_PA_^c^	Yield^d^ (g)	Activity (Kg/g-cat.)	*M*_n_^e^	*M*_w_/*M_n_*^e^	*T*_g_^f^ (°C)

1	1.0	0	1.5	38	0.12	(2.5)	(0.83)	115,000	1.22	29, 69
2	1.0	0	2.0	68	0.23	(2.5)	(0.83)	215,000	1.33	48
3	1.0	0	2.5	91	0.15	(4.7)	(1.6)	198,000	1.40	44
4	1.0	0	3.0	100	0.11	6.5 (7.0)	2.2	381,000	1.27	41
5	2.0	0	1.5	31	0.31	(1.8)	(0.60)	87,000	1.20	24, 73
6	2.0	0	2.0	86	0.39	(4.2)	(1.4)	193,000	1.22	43
7	2.0	0	3.0	100	0.23	7.3 (7.4)	2.4	354,000	1.23	48
8	1.0	5.0	1.5	50	0.18	(2.2)	(0.73)	19,000	1.08	25, 67
9	1.0	10	1.5	59	0.19	(2.6)	(0.87)	10,000	1.06	25, 72
10	1.0	15	1.5	63	0.22	(2.4)	(0.80)	8,000	1.07	25, 69
11	1.0	20	1.5	63	0.25	(2.2)	(0.73)	5,000	1.05	14
12	1.0	15	2.0	80	0.18	(3.6)	(1.2)	11,000	1.03	28
13	1.0	15	2.5	100	0.13	5.7 (5.9)	1.9	16,000	1.03	39
14	1.0	15	3.0	100	0.12	6.4 (6.3)	2.1	19,000	1.03	39
15	1.0	15	4.0	100	0.10	7.0 (7.6)	2.3	26,000	1.04	38
16	2.0	15	3.0	100	0.24	6.6 (7.2)	2.2	22,000	1.05	43

^a^Polymerization conditions: PO (10.4 g, 180 mmol), catalyst **1** (3.0 mg, 1.8 μmol), CO_2_ (35 bar), 80 °C. ^b^Including heating time of ca. 50 min. ^c^Mole fraction of PA in the polymers determined by ^1^H NMR spectroscopy. ^d^Values in parentheses calculated from conversion and *f*_PA_. ^e^Determined on GPC using a polystyrene standard. ^f^Determined on DSC.

Catalyst residues could also be removed completely by filtration through a short pad of silica gel to provide colorless polymers (see Supporting Information File 1). The catalyst residue should be removed thoroughly because it is not only toxic, but also leads to the thermal instability of the products [32]. It was not easy to separate the generated polymer and the unreacted PA; the polymers were obtained admixed with the unreacted PA unless 100% conversion of PA was reached.

The pattern of the aromatic signals in the ^1^H NMR spectra was different from that observed for the alternating PO/PA copolymer: just two broad signals were observed at 7.71 and 7.57 ppm (Figure 1(B)). Four OC*H*(Me) signals were observed at 5.45, 5.30, 5.10, and 4.95 ppm. The large signal at 4.95 ppm (marked "d" in Figure 1(B)) was assigned unambiguously to the carbonate-C*H*(Me)CH_2_-carbonate signal through comparison with the spectrum of PPC (Figure 1(C)). The signals at 5.30 ppm (marked "b") and 5.10 ppm (marked "c") were assigned to ester-C*H*(Me)CH_2_-carbonate and ester-CH_2_C*H*(Me)-carbonate, respectively. A comparatively small signal was observed at 5.45 ppm (marked "a"), which was assigned to ester-C*H*(Me)CH_2_-ester. The mole fractions of PA in the polymers (*f*_PA_) were determined from the ^1^H NMR spectra using the equation *f*_PA_ = [(*I*_7.5-7.8_)/4]/*I*_4.8-5.4_, where *I*_7.5-7.8_ and *I*_4.8-5.4_ are the integrated values in the region 7.5–7.8 ppm (benzene-*H* signal) and 4.8–5.4 ppm (OC*H*Me signal), respectively. The yields calculated from the conversion (*c*) and *f*_PA_ [yield (g) = (206.19 × *y* + 102.08 × *y* × (1 − *f*_PA_)/*f*_PA_), where *y* is the number of moles of consumed PA, that is, *y* = (fed PA (g) × *c*)/148.12], were in good agreement with the measured weights of the isolated polymers at full PA conversion (Table 2, entries 4, 7, 13–16). The *f*_PA_ values decreased gradually from 0.23 to 0.11 upon increasing the reaction time from 2.0 to 3.0 h (Table 2, entries 2–4). The CO_2_ concentration was almost unchanged at the pressure of 35–33 bar, whereas the free PA concentration in solution was gradually depleted, resulting in lower *f*_PA_ values with increased polymerization times. A deviation was observed at a very early reaction time of 1.5 h (Table 2, entry 1), where the *f*_PA_ value was low (0.12) even at a high PA concentration. This deviation might be attributed to the uncontrolled reaction temperature. The bomb reactor was warmed slowly using a hot oil bath (80 °C), reaching the desired temperature (80 °C) in ca. 50 min. If the small portion of chains grown during the warming time is ignored, the generated polymers should be gradient poly(1,2-propylene carbonate-*co*-phthalate). The chains grown in the early stages were enriched with PA units, while those grown at a later stage were enriched with or composed solely of carbonate units. When the fed PA amount was doubled to 2.0 g, polymers with higher PA mole fractions were generated (Table 2, entries 5–7). Upon running the reaction for 3.0 h, full conversion of PA was also achieved (Table 2, entry 7). The *f*_PA_ value at this full conversion was 0.23, which was twice that attained at the PA feeding of 1.0 g. Related stereogradient CO_2_/PO copolymers have been reported, in which one end of the polymer chain is enriched with the *R*-isomer of PO, while the other is enriched with the *S*-isomer [33–34]. A related copolymer composed of aliphatic polycarbonate and aromatic polyester units [poly(1,4-butylene terephthalate-*co*-carbonate)], prepared by condensation polymerization of 1,4-butanediol, dimethyl terephthalate, and dimethyl carbonate, has also been reported recently [35–36].

When ethanol (5.0 mg, 60 equiv/**1**) was fed as a chain-transfer agent with 1.0 g PA, faster PA consumption was observed (Table 2, entry 8). At the initial stage of 1.5 h including a heating time of ca. 50 min, the PA conversion was 50% with a high *f*_PA_ value (0.18) compared with that in the absence of ethanol (PA conversion, 38%; *f*_PA_, 0.12). The PA conversions at the identical reaction time of 1.5 h increased gradually from 50% to 63% upon increasing the ethanol feeding amount from 5.0 to 20 mg, and the *f*_PA_ values also increased gradually from 0.18 to 0.25 (entries 8–11). Because of the faster PA consumption, full PA conversion was achieved in 2.5 h (entry 13). At this full conversion, the polymerization solution was stirrable because of the formation of a low-molecular-weight polymer. By running the polymerization for a further 1.0 or 2.0 h, the yields increased further from 5.7 g to 6.4 and 7.0 g, respectively (entries 14, 15). During the additional polymerization time after full PA conversion, only the carbonate units were grown, resulting in the formation of a block copolymer. One side of the block copolymer was gradient poly(1,2-propylene phthalate-*co*-carbonate), while the other side was poly(1,2-propylene carbonate). When 2.0 g of PA was fed along with 15 mg of ethanol, full conversion of PA was achieved in 3.0 h and the *f*_PA_ value (0.24) was almost twice that attained at a PA feed of 1.0 g (entry 16).

**Figure 3:**
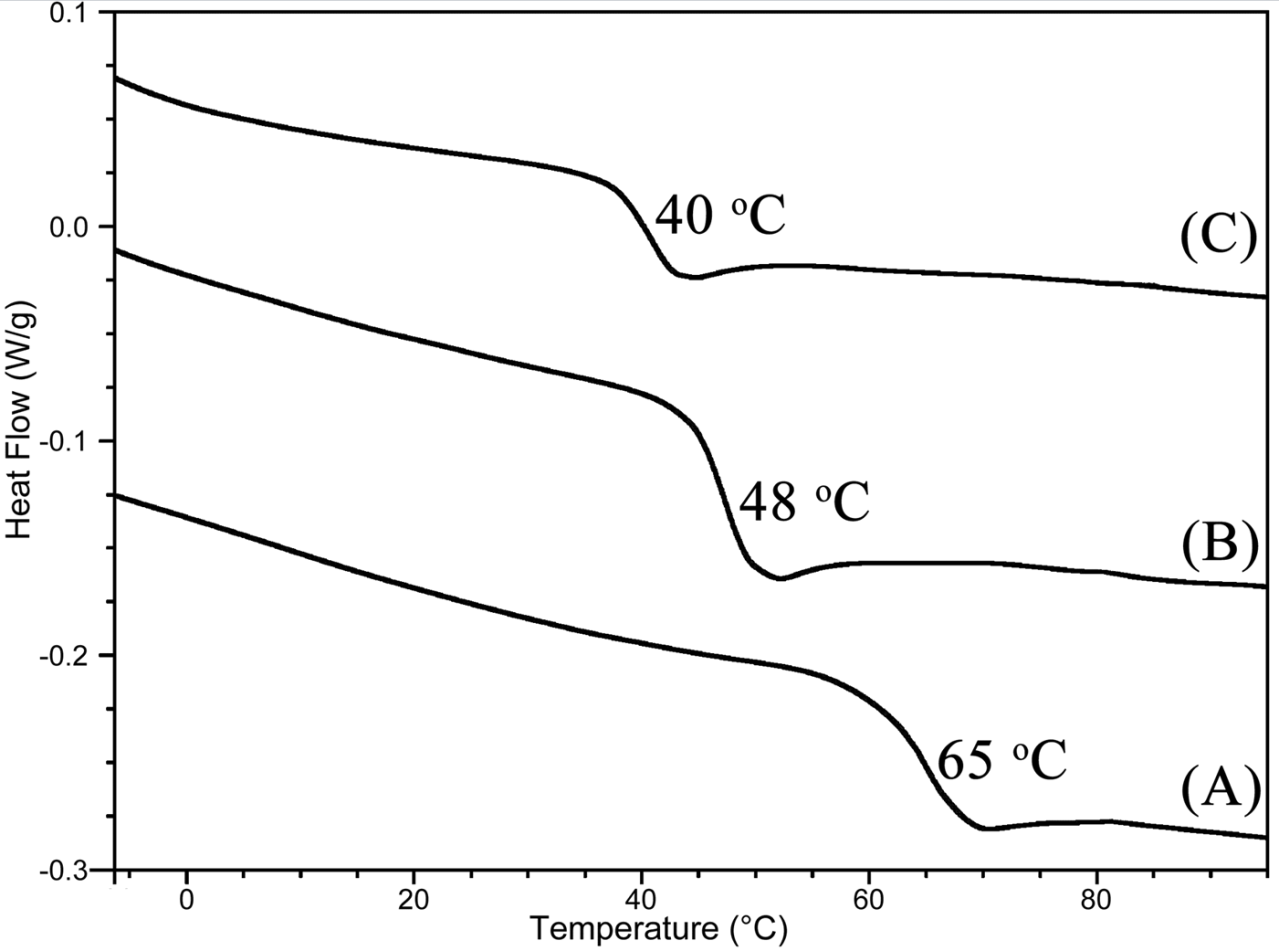
DSC curves of PO/PA alternating polymer (**A**, entry 1 in Table 1), PO/CO_2_/PA terpolymer (**B**, entry 7 in Table 2), and PO/CO_2_ alternating copolymer (**C**).

When a substantial number of PA units (*f*_PA_, 0.23) was incorporated in PO/CO_2_/PA terpolymers, a high *T*_g_ of 48 °C was observed (entries 2 and 7), which was higher than that of the PO/CO_2_ alternating copolymer (40 °C), but lower than that of the PO/PA alternating polymer (65 °C) (Figure 3). With a small number of incorporated PA units (*f*_PA_, ca. 0.1), *T*_g_ was similar to that of the PO/CO_2_ alternating copolymer (entries 4, 13–15). For the polymers generated at the early stage (1.5 h), two *T*_g_ signals were observed at 25 °C and ca. 70 °C (entries 1, 5, 8–10).

In the polymerization reaction, the nitrate and acetate anions in **1** became chain-growing carbonate or alkoxide anions [14,17]. In the presence of protic compounds such as water (present as an impurity) or alcohols (deliberately added), reversible proton exchange reactions occur rapidly between the chain-growing anions and protic compounds, resulting in uniform chain growth not only from the protic compounds but also from the anions in **1**. In all cases, when the polymerizations were carried out without the deliberate addition of a chain-transfer agent (Table 2, entries 1–7), bimodal GPC curves were observed (see Supporting Information File 1). Polymer chains in the high-molecular-weight mode were grown biaxially from water, while those in the low-molecular-weight mode were grown from the anions in **1**. The ratio of the two modes was different for each entry. A very small amount of catalyst **1** was fed under the polymerization conditions employed (**1**/PO = 1:100,000), so the number of impurity water molecules was not negligible, and varied in the range of the same order of the number of anions in **1**, even with thorough drying of PO and CO_2_. High-molecular-weight polymers with *M*_n_ 381,000 and 354,000 were obtained at the full conversions of 1.0 g and 2.0 g PA, respectively (Table 2, entries 4 and 7). The molecular weights were controlled by the amount of ethanol fed as a chain-transfer agent. At the feeding amount of 15 mg of ethanol (180 equiv per **1**) and full conversion of PA (Table 2, entries 13–16), low-molecular-weight polymers with *M*_n_ 16,000–26,000 were generated. The numbers of polymer chains generated per **1** under those conditions were calculated to be 198, 187, 145, and 167, respectively, roughly in agreement with the number of ethanol molecules (180 equiv per **1**). For the feeding of a large amount of ethanol (180 equiv per **1**), the number of polymer chains grown biaxially from the water molecules was negligible, and very narrow unimodal distributions were observed with *M*_w_/*M*_n_ of ca. 1.05 in the GPC curves.

**Scheme 4:**
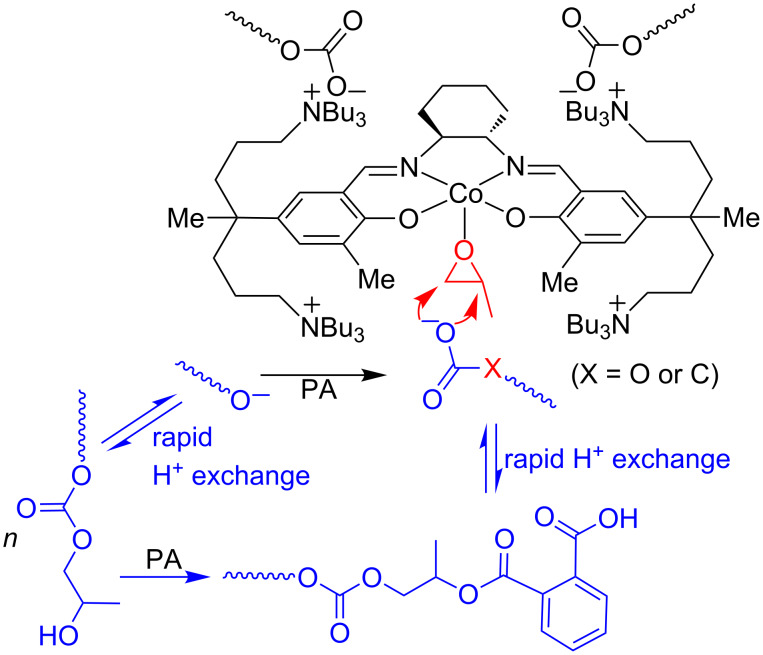
PA incorporation process in PO/CO_2_/PA terpolymerization.

Scheme 4 shows the PA incorporation process in the PO/CO_2_/PA terpolymerizations. In addition to the direct attack of the alkoxide anion on PA, another PA consumption process might operate in the presence of the chain transfer agent such as deliberated added ethanol or impurity water. In this process, PA directly reacted with the formed OH chain terminus, leading to incorporation of PA without the action of the catalyst. This process made the PA conversion faster, consequently helping to achieve the full conversion of PA.

## Conclusion

The (salen)Co(III) complex **1** tethering four quaternary ammonium salts, which is a highly active catalyst for CO_2_/PO copolymerization, worked efficiently as a catalyst in both PO/PA copolymerizations and CO_2_/PO/PA terpolymerizations. An attractive feature in view of commercial application is that full conversion of PA could be achieved within 5 h in both polymerizations, even under the conditions of a high feeding amount of PA (2.0 g PA/10 g PO) and a very small feeding amount of catalyst **1** (3.0 mg). No ether linkages were formed in either of the polymerizations to afford poly(1,2-propylene phthalate) or poly(1,2-propylene phthalate-*co*-carbonate). The latter had a gradient composition with one end enriched with PA units and the other enriched with carbonate units. Both showed immortal polymerization character: the molecular weights of the resulting polymer were controlled precisely by the activity (g/mol-**1**) and the number of chain-growing sites per **1** [anions in **1** (5) + water (present as impurity) + ethanol (deliberately fed)], and the molecular weight distributions were narrow (*M*_w_/*M*_n_, 1.05–1.5). Because of the high activity of **1**, polymers with very high molecular weights were generated (*M*_n_ up to 170,000 and 350,000 for PO/PA copolymerization and CO_2_/PO/PA terpolymerization, respectively). The terpolymer bearing a substantial number of PA units (*f*_PA_, 0.23) showed a higher *T*_g_ value (48 °C) than the CO_2_/PO alternating copolymer (40 °C).

## Experimental

**General remarks.** CO_2_ gas (99.999% purity) was dried through storage in a column of molecular sieves 3Å at a pressure of 30 bar. PO was dried by stirring over CaH_2_ and then vacuum-transferred to a reservoir. PA was purchased from Aldrich and purified by recrystallization in ethyl acetate. ^1^H NMR (400 MHz) and ^13^C NMR (100 MHz) spectra were recorded on a Varian Mercury Plus 400 instrument. Gel permeation chromatography (GPC) was performed in THF at 40 °C using a Waters Millennium apparatus with polystyrene standards. The *T*_g_ data were determined from a second heating at a heating rate of 10 °C/min by differential scanning calorimetry (DSC) using a Thermal Analysis Q10 instrument.

**Typical procedure for PO/PA alternating copolymerizations.** A bomb reactor (ca. 50 mL) was assembled inside a glove box after being charged with **1** (3.0 mg, 1.8 μmol), PO (10.4 g, 179 mmol), and PA (and ethanol as a chain-transfer agent). The reactor was immersed in a hot oil bath (80 °C). After running of the polymerization for a given time, the reactor was cooled to room temperature. An aliquot was taken to measure the PA conversion by ^1^H NMR spectroscopy. The polymer solution was filtered over a short pad of silica gel to remove the catalyst residue. The silica gel pad was washed with methylene chloride (10 mL × 2). The colorless filtrates were combined. The solvent was removed using a rotary evaporator, and the residual solvent was removed completely by keeping the isolated lump in a vacuum oven overnight at 100 °C. When the PA conversion was not 100%, the polymer was obtained admixed with unreacted PA. For DSC studies, small pieces of the polymer lump admixed with the unreacted PA were dissolved in a copious amount of CH_2_Cl_2_, and the resulting solution was eluted through a relatively long pad of silica gel.

**Typical procedure for PO/CO_2_/PA terpolymerizations.** A bomb reactor (ca. 50 mL) was assembled inside a dry box after being charged with **1** (3.0 mg, 1.8 μmol), PO (10.4 g, 179 mmol), and PA (and ethanol as a chain-transfer agent). The CO_2_ gas was pressurized to 25 bar at room temperature, and the reactor was then immersed in a hot oil bath (80 °C). When the temperature inside the bomb reactor reached the bath temperature, the pressure was 35 bar. After running of the polymerization for a given time, the reactor was cooled to room temperature through immersion in an ice bath. CO_2_ gas was released and the reactor was opened. An aliquot was taken to measure the PA conversion by ^1^H NMR spectroscopy. The catalyst removal and work-up procedures were as described for the PO/PA alternating copolymerizations.

## Supporting Information

File 1 ^1^H NMR spectra, ^13^C NMR spectra, GPC curves, and the pictures of the isolated polymers in PO/PA alternating polymerizations and PO/CO_2_/PA terpolymerizations.
