# Supplementary material for: Copolymerization and terpolymerization of carbon dioxide/propylene oxide/phthalic anhydride using a (salen)Co(III) complex tethering four quaternary ammonium salts
Source: Beilstein J Org Chem. 2014 Aug 5;10:1787–95. doi: 10.3762/bjoc.10.187 (PMC4142976; doi:10.3762/bjoc.10.187)
Supplement: File 1 — 1H NMR spectra, 13C NMR spectra, GPC curves, and the pictures of the isolated polymers in PO/PA alternating polymerizations and PO/CO2/PA terpolymerizations. [file Beilstein_J_Org_Chem-10-1787-s001.pdf]

Supporting Information

for

Copolymerization and terpolymerization of carbon dioxide/propylene oxide/phthalic anhydride using a (salen)Co(III) complex tethering four quaternary ammonium salts

Jong Yeob Jeon, Seong Chan Eo, Jobi Kodiyan Varghese, and Bun Yeoul Lee*

Address: Department of Molecular Science and Technology, Ajou University, Suwon 443-749 Korea

Email: Bun Yeoul Lee - bunyeoul@ajou.ac.kr

* Corresponding author

^{1}H NMR spectra, ^{13}C NMR spectra, GPC curves, and the pictures of the isolated polymers in PO/PA alternating polymerizations and PO/CO_2/PA terpolymerizations.

<^{1}H NMR spectrum of PO/PA alternating copolymer prepared using 2.0 g PA for 3.0 h polymerization (entry 2 in Table 1)>

The signals marked with “*” and “#” are the THF-d_8 and the residual PO signals, respectively.

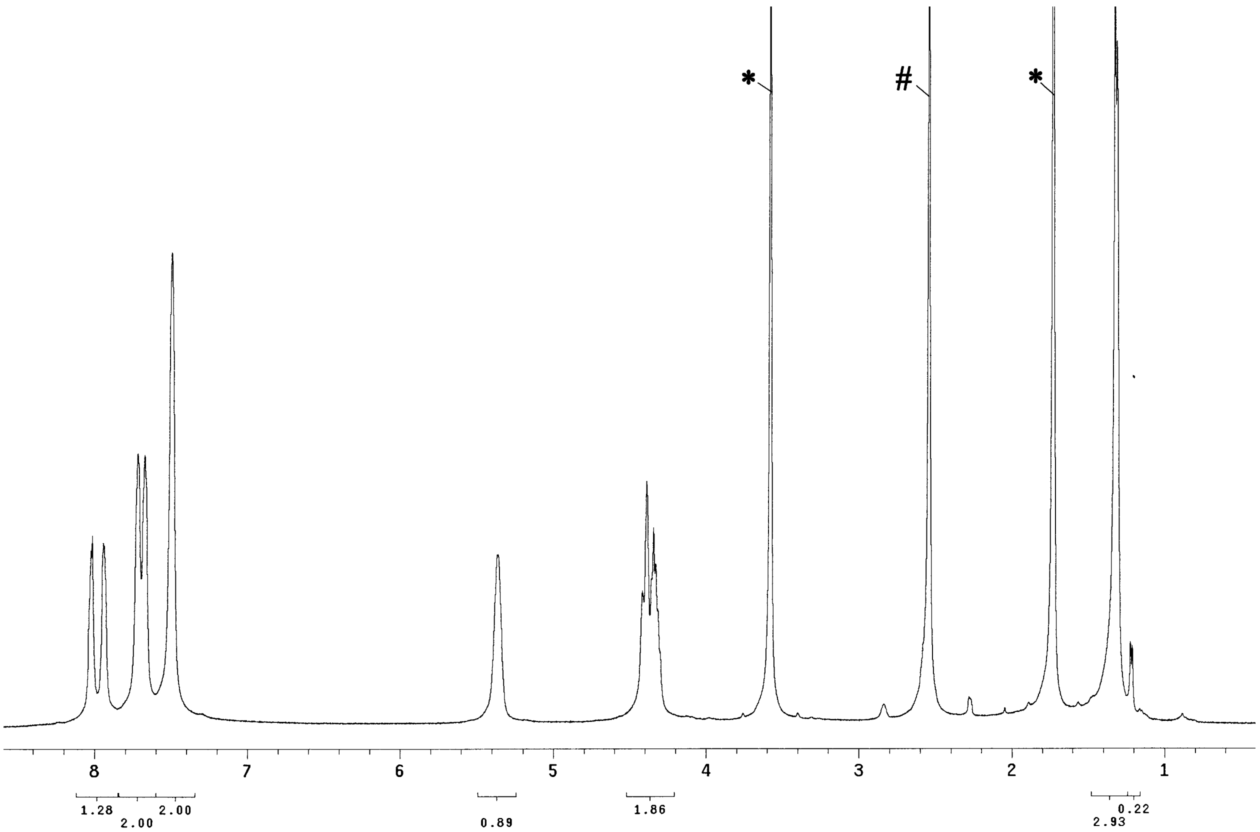

<^{1}H NMR spectrum of CO_2/PO/PA terpolymer prepared using 1.0 g PA for 1.5 h (entry 1 in Table 2)>

The signals marked with "*" and "#" are ones of THF-d_8 and the residual PO, respectively

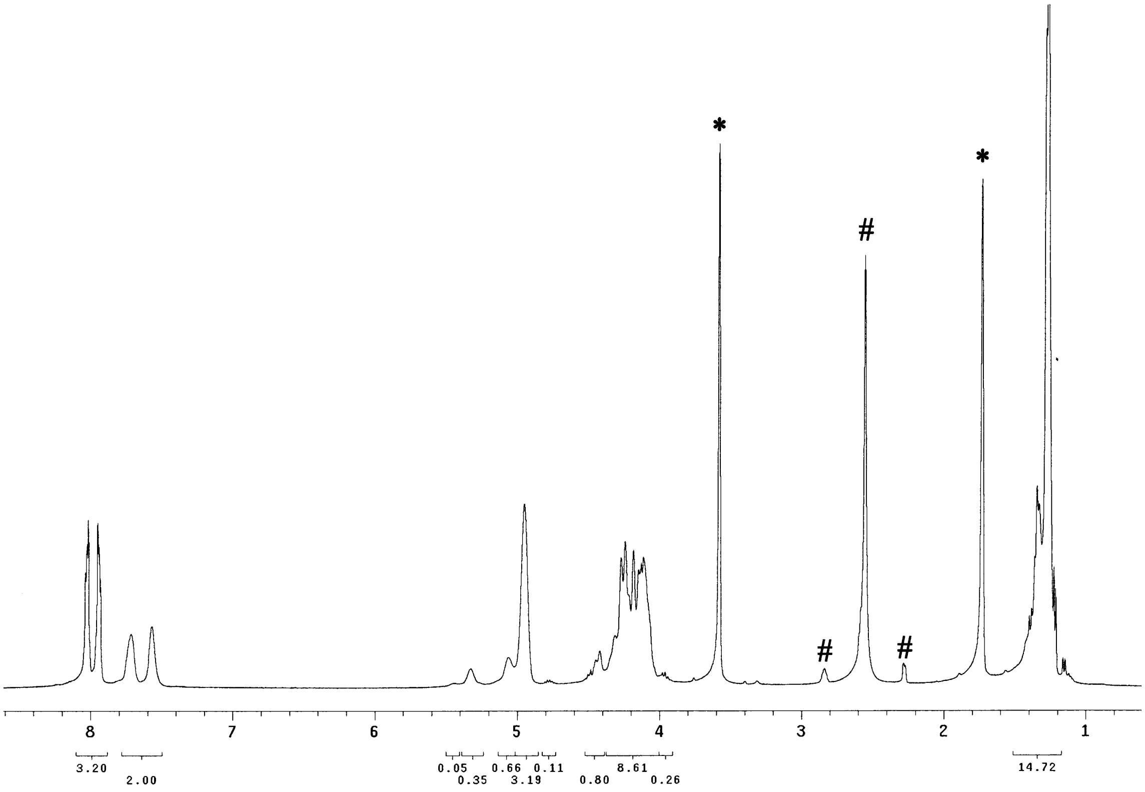

<^{1}H NMR spectrum of CO_2/PO/PA terpolymer prepared using 1.0 g PA for 2.0 h (entry 2 in Table 2)>

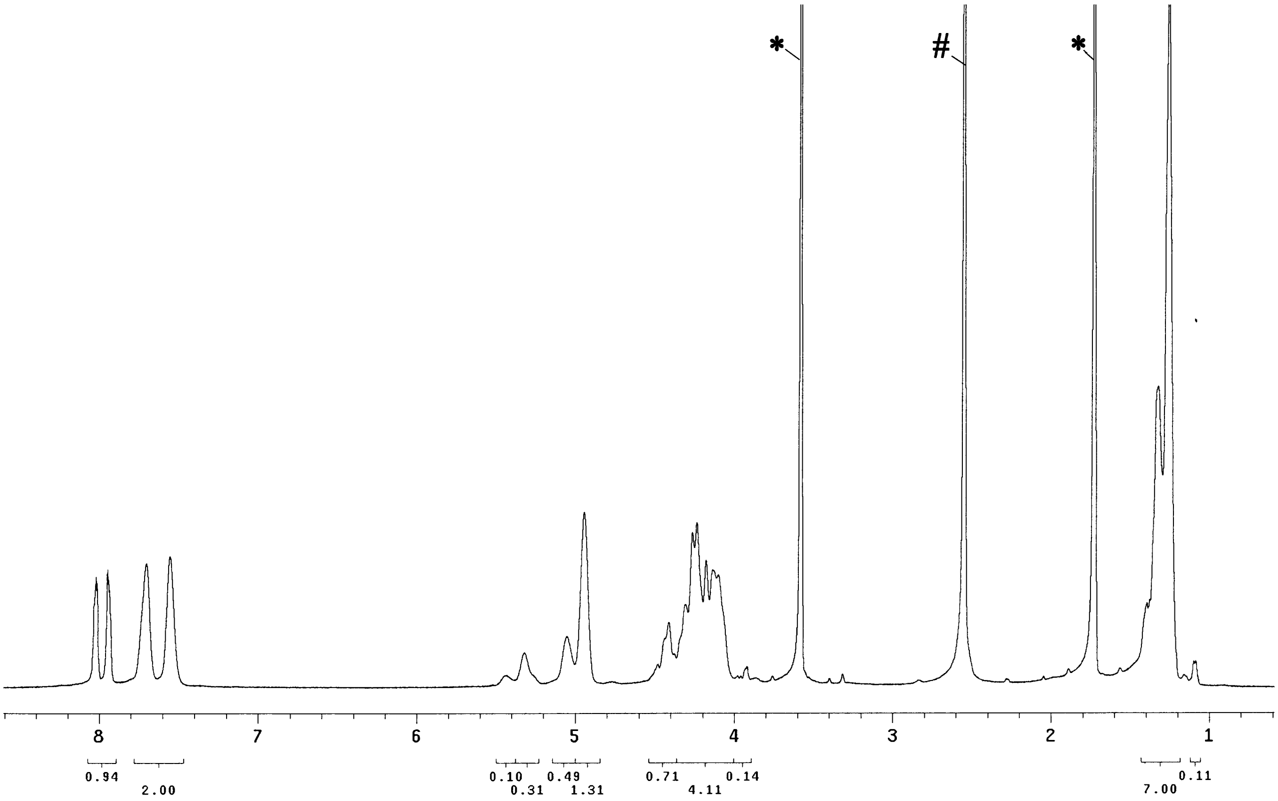

<^{1}H NMR spectrum of CO_2/PO/PA terpolymer prepared using 1.0 g PA for 2.5 h (entry 3 in Table 2)>

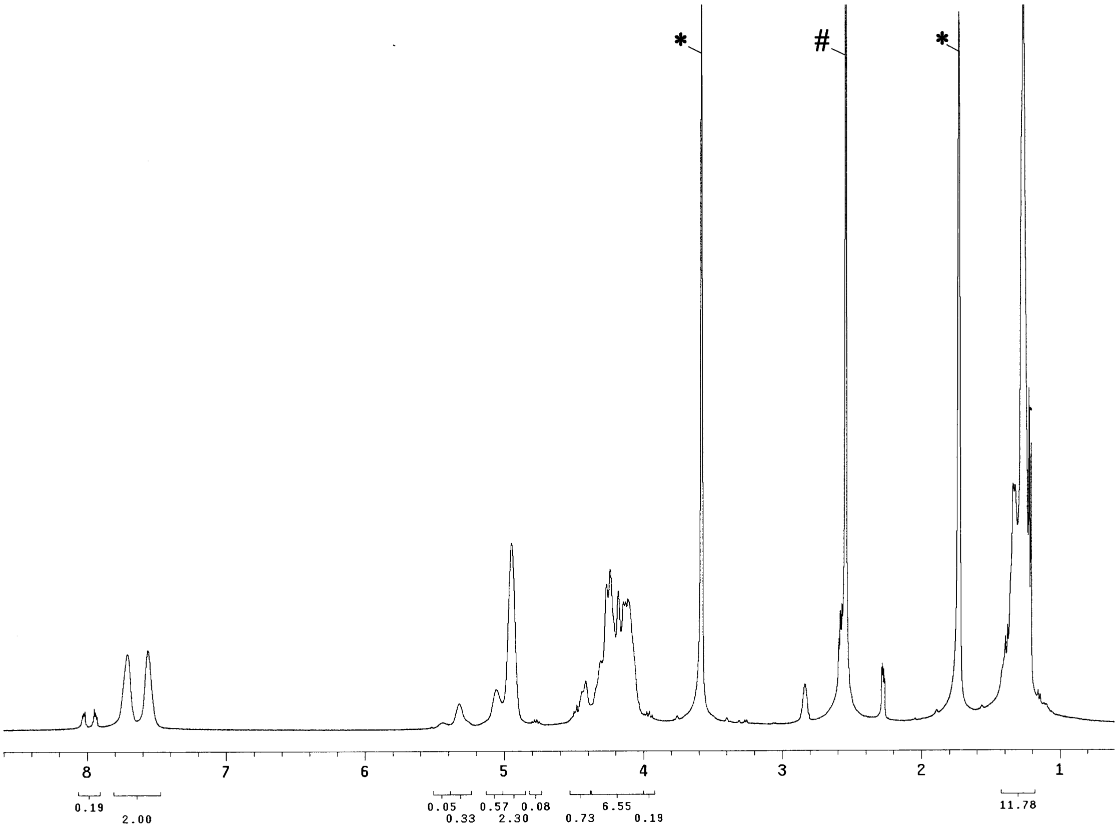

<^{1}H NMR spectrum of CO_2/PO/PA terpolymer prepared using 1.0 g PA for 3.0 h (entry 4 in Table 2)>

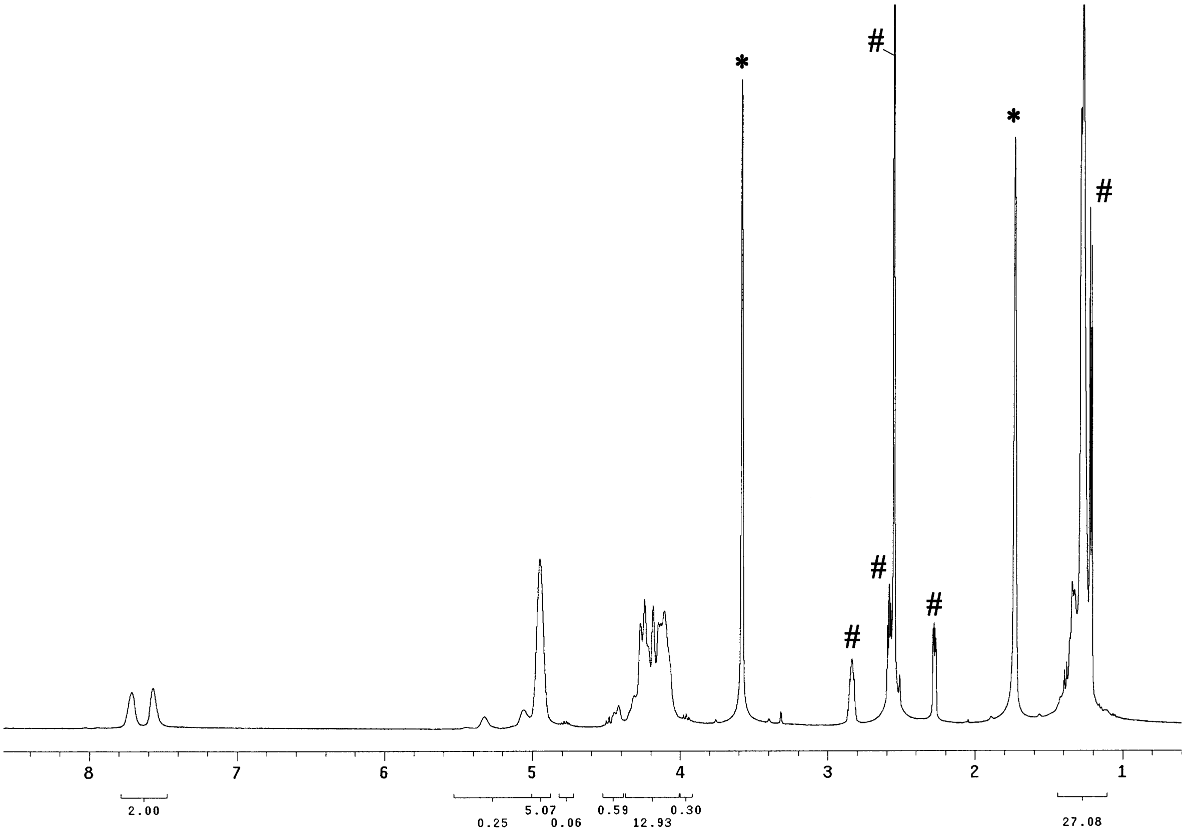

<^{1}H NMR spectrum of CO_2/PO/PA terpolymer prepared using 2.0 g PA for 1.5 h (entry 5 in Table 2)>

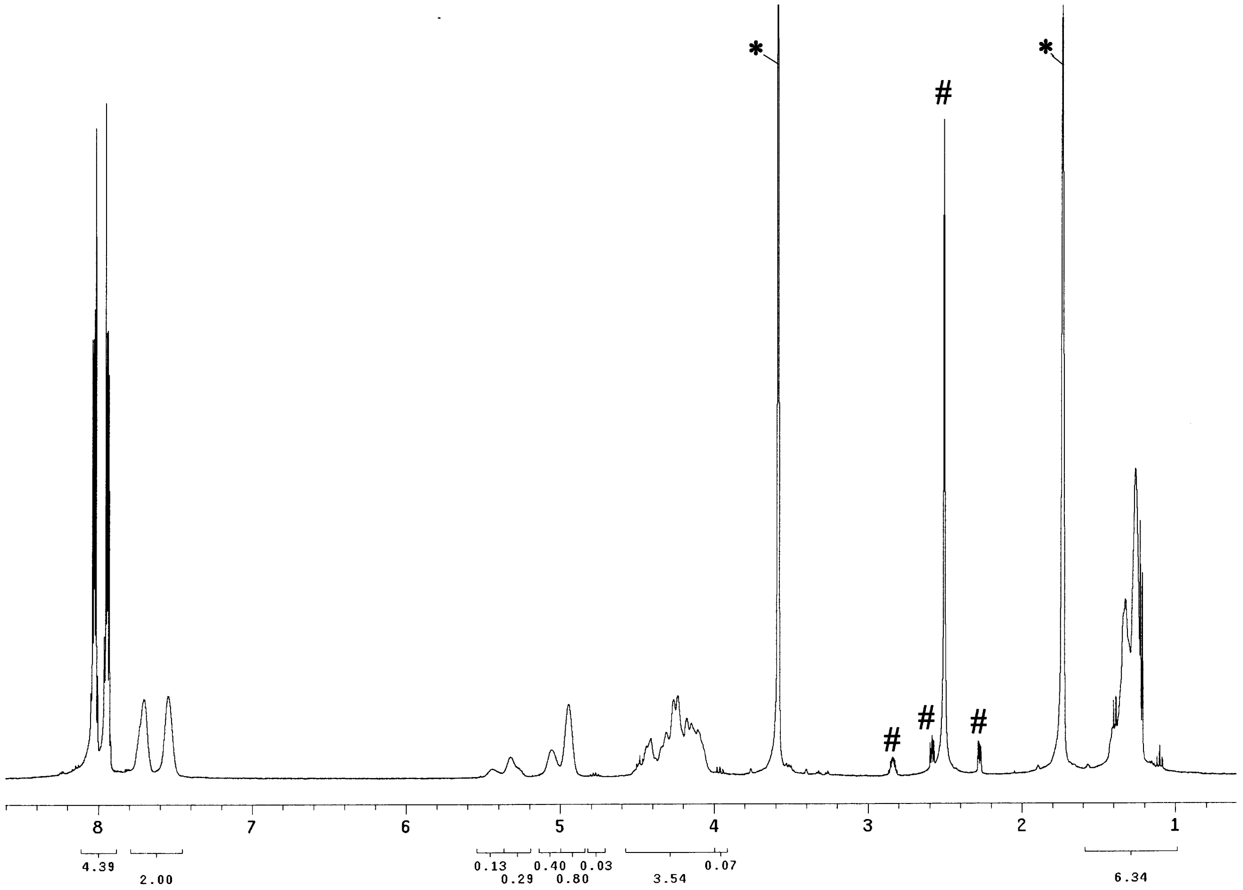

<^{1}H NMR spectrum of CO_2/PO/PA terpolymer prepared using 2.0 g PA for 2.0 h (entry 6 in Table 2)>

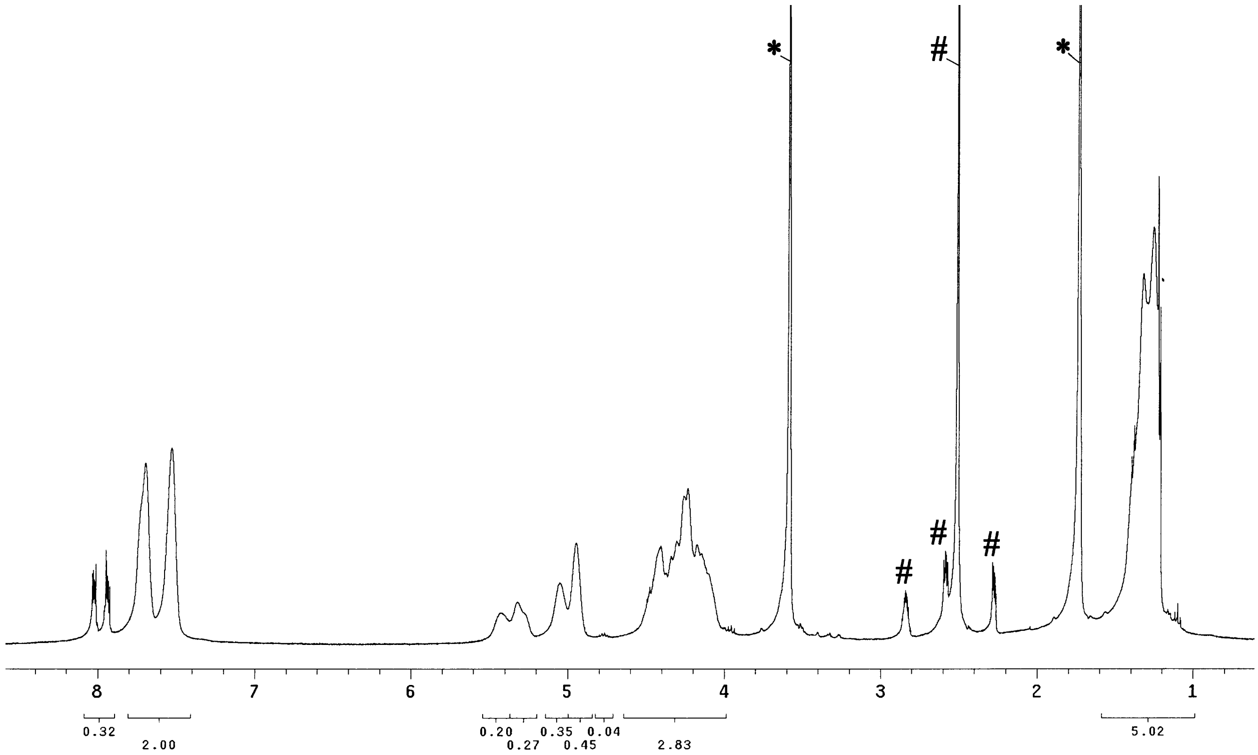

<^{1}H NMR spectrum of CO_2/PO/PA terpolymer prepared using 2.0 g PA for 3.0 h (entry 7 in Table 2)>

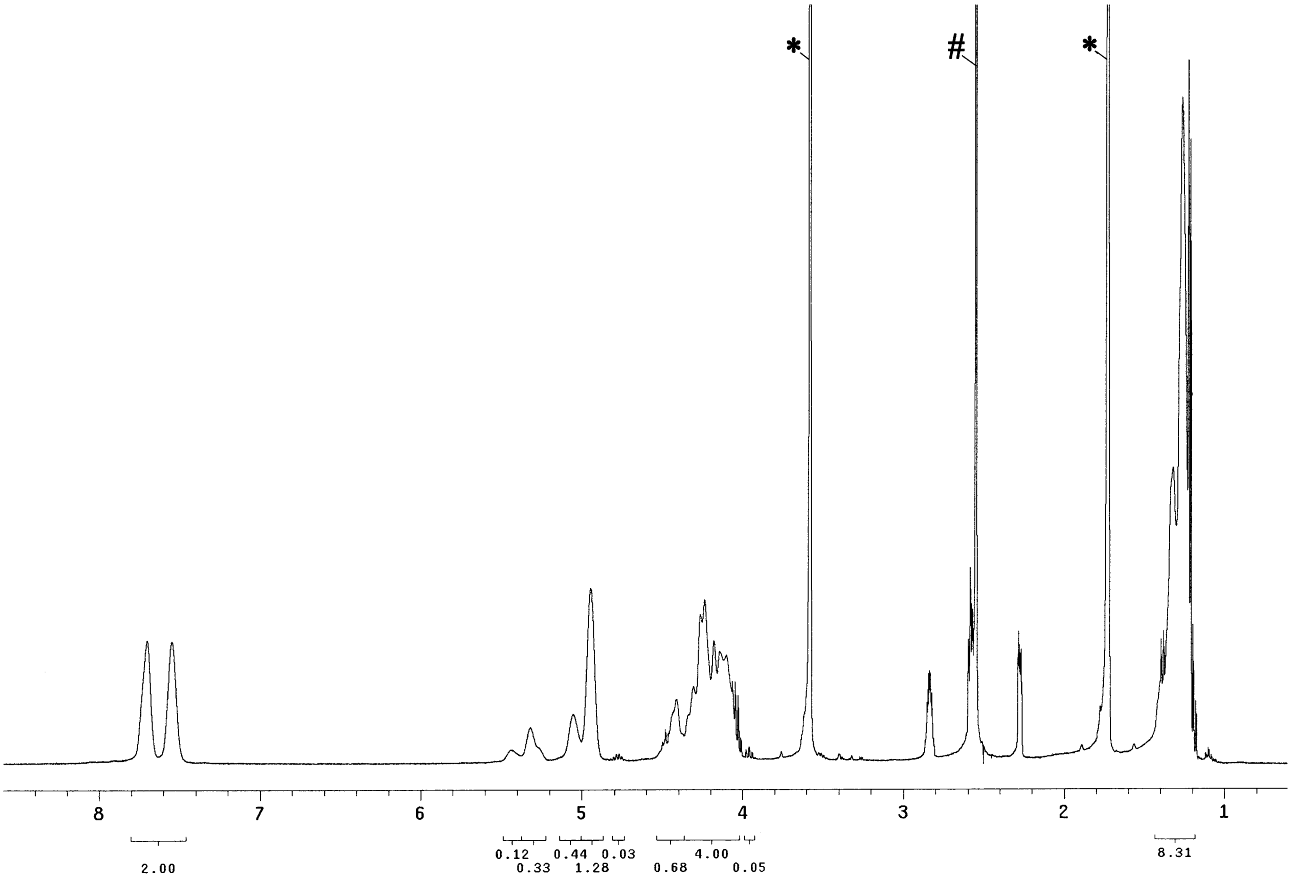

<^{1}H NMR spectrum of CO_2/PO/PA terpolymer prepared using 1.0 g PA and ethanol 5.0 mg for 1.5 h (entry 8 in Table 2)>

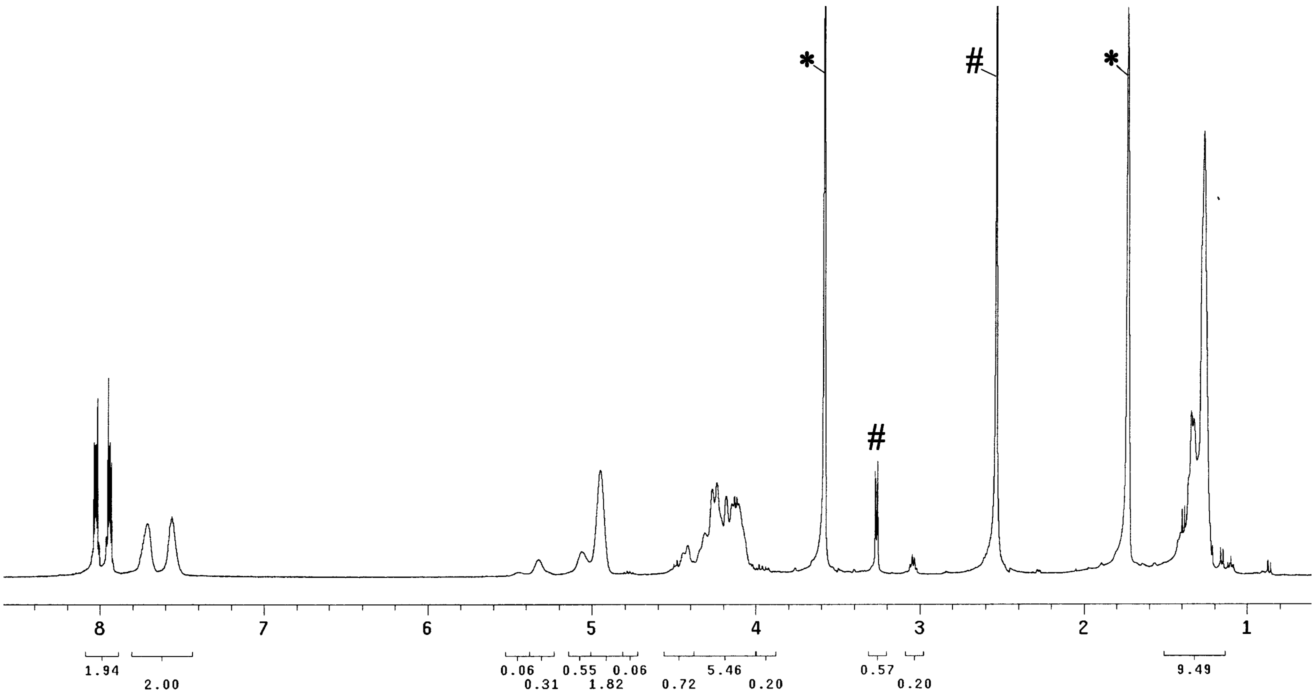

<^{1}H NMR spectrum of CO_2/PO/PA terpolymer prepared using 1.0 g PA and ethanol 10 mg for 1.5 h (entry 9 in Table 2)>

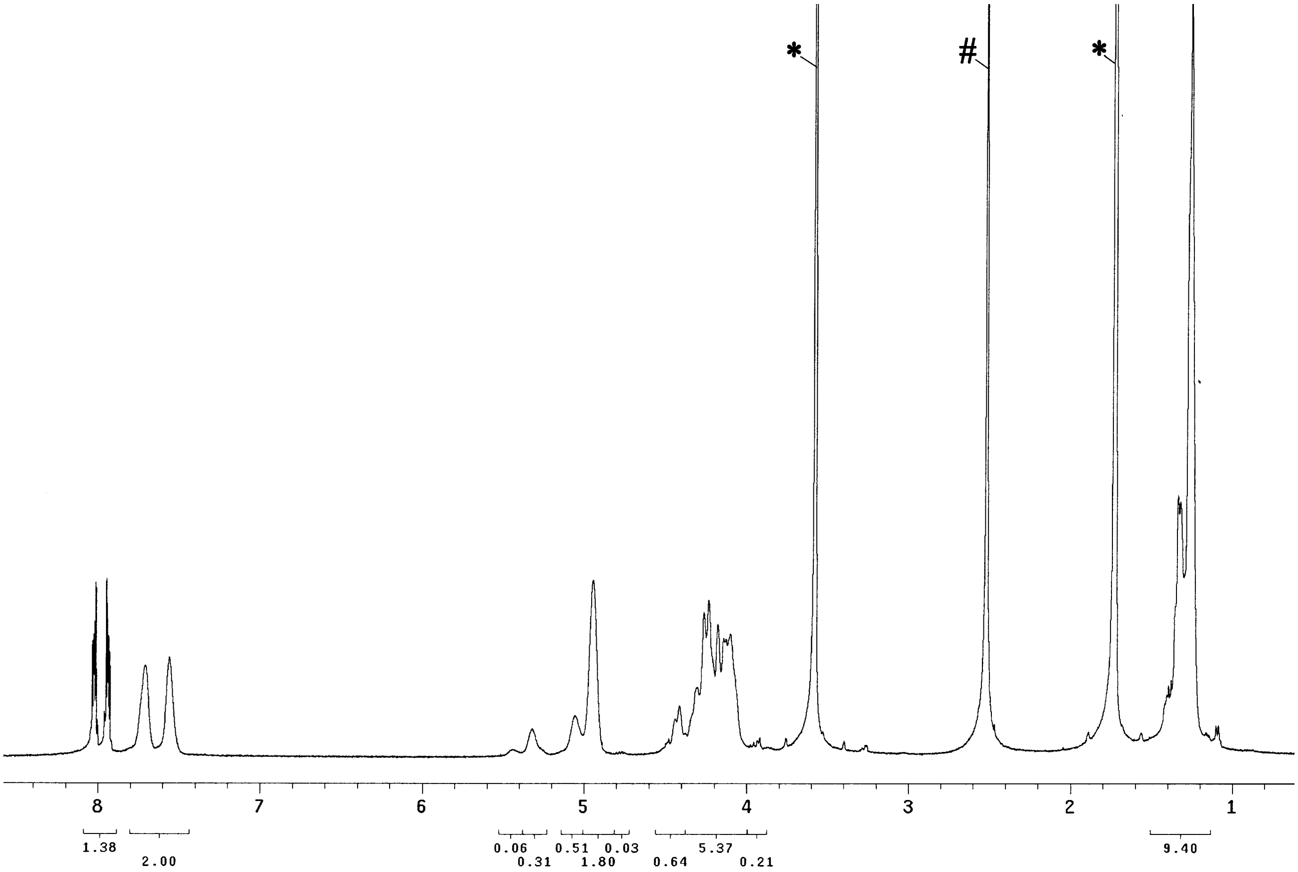

<^{1}H NMR spectrum of CO_2/PO/PA terpolymer prepared using 1.0 g PA and ethanol 15 mg for 1.5 h (entry 10 in Table 2)>

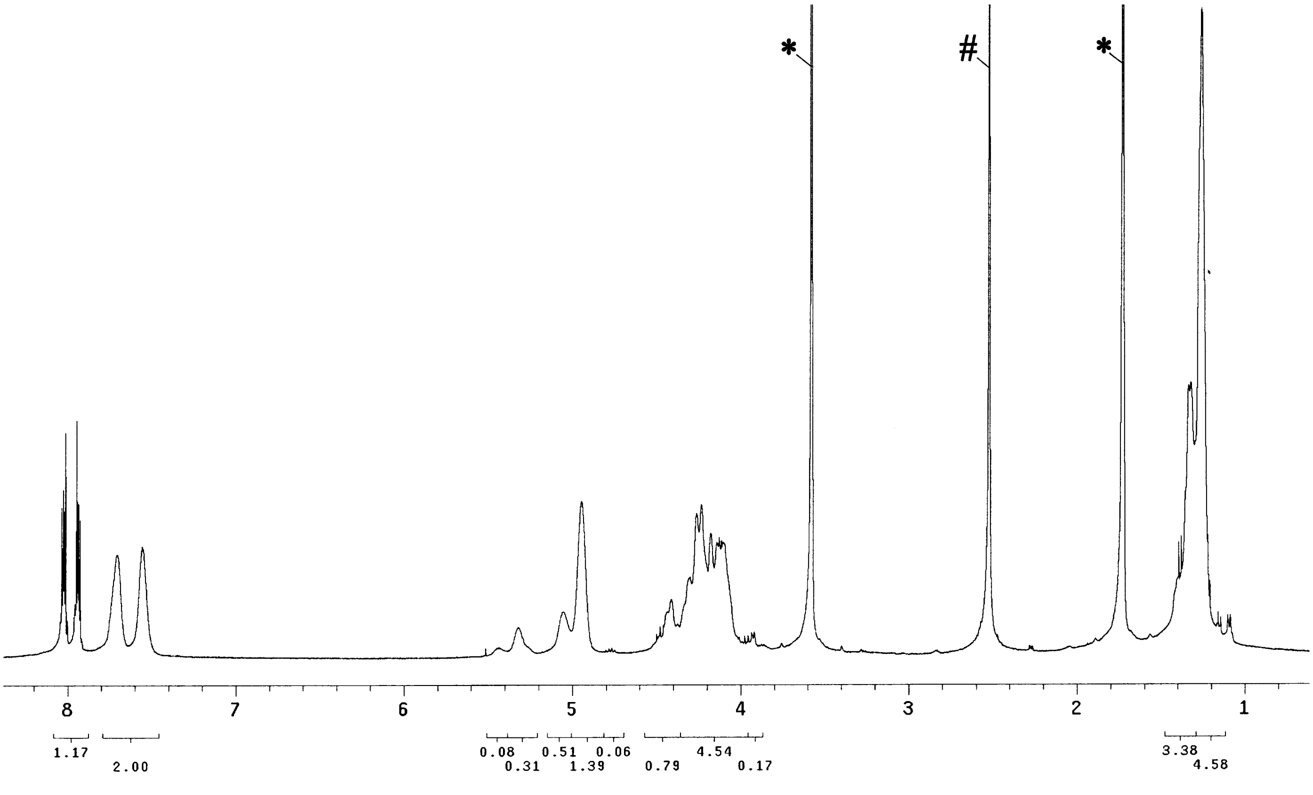

<^{1}H NMR spectrum of CO_2/PO/PA terpolymer prepared using 1.0 g PA and ethanol 20 mg for 1.5 h (entry 11 in Table 2)>

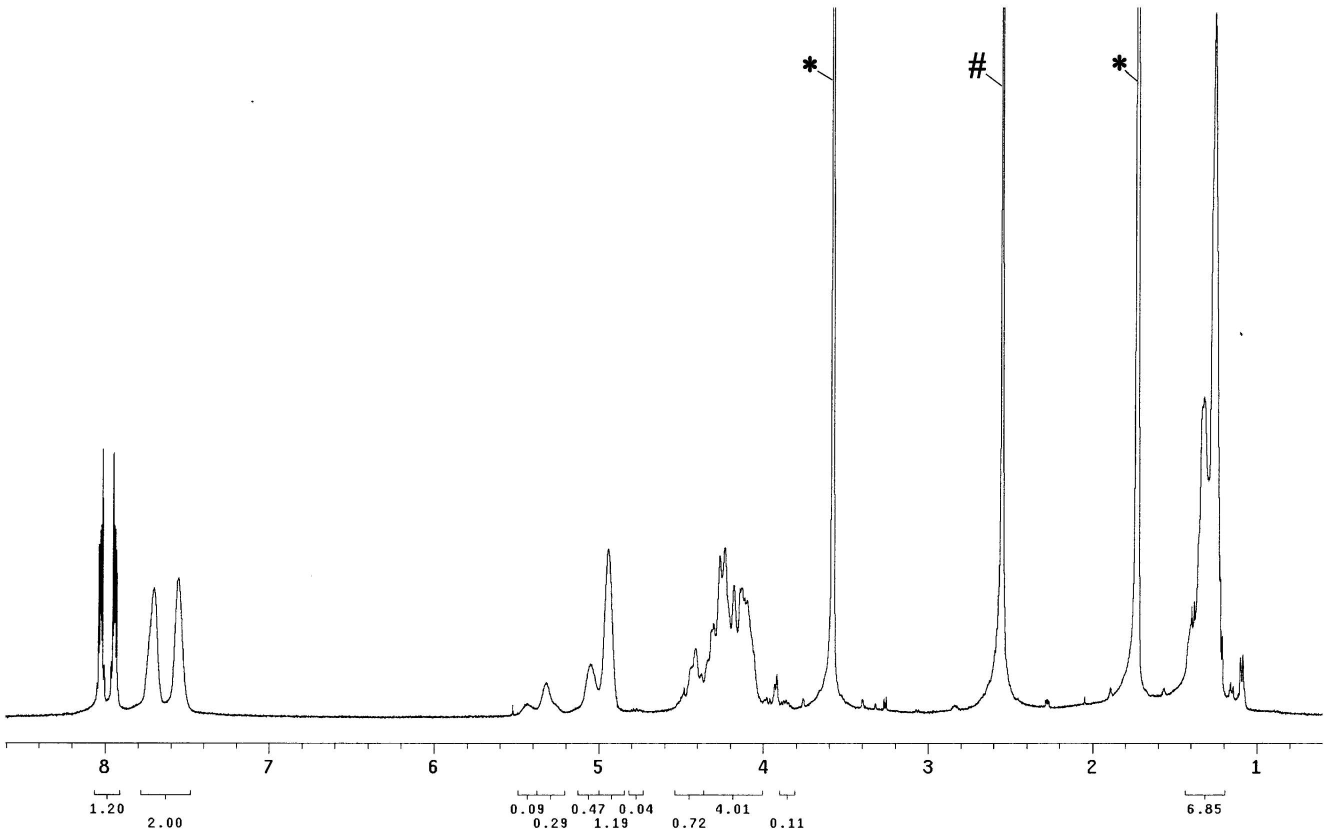

<^{1}H NMR spectrum of CO_2/PO/PA terpolymer prepared using 1.0 g PA and ethanol 15 mg for 2.0 h (entry 12 in Table 2)>

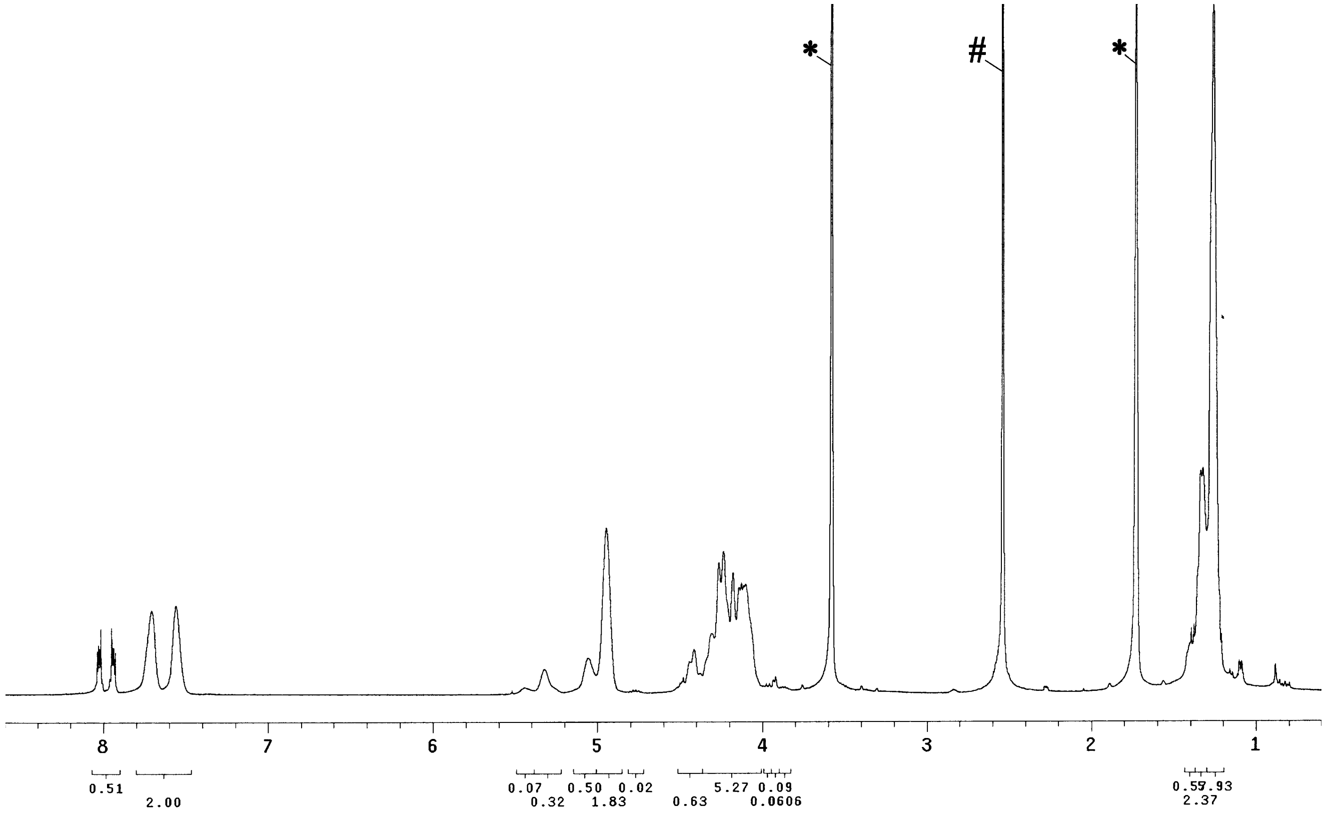

<^{1}H NMR spectrum of CO_2/PO/PA terpolymer prepared using 1.0 g PA and ethanol 15 mg for 2.5 h (entry 13 in Table 2)>

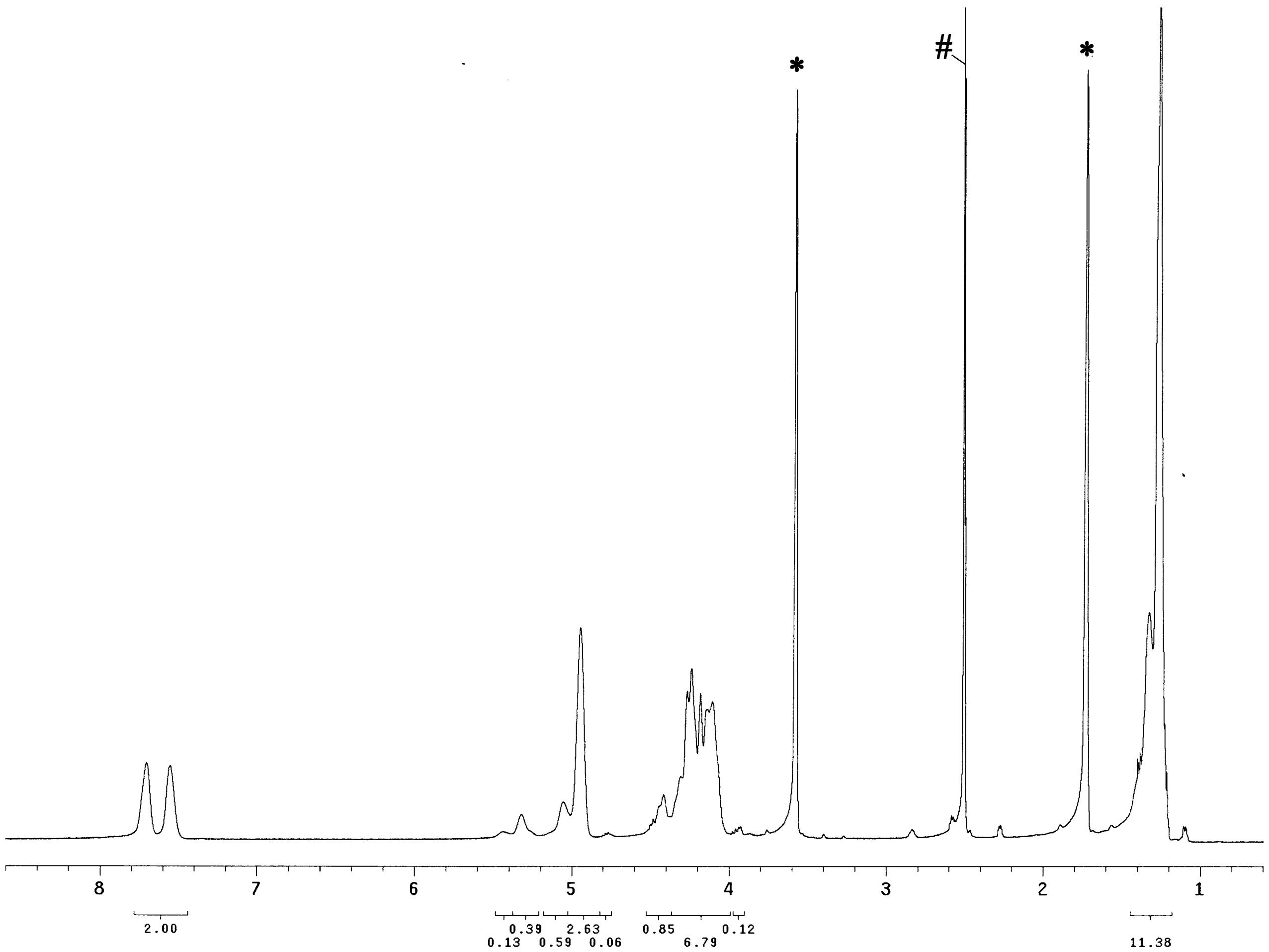

<^{1}H NMR spectrum of CO_2/PO/PA terpolymer prepared using 1.0 g PA and ethanol 15 mg for 3.0 h (entry 14 in Table 2)>

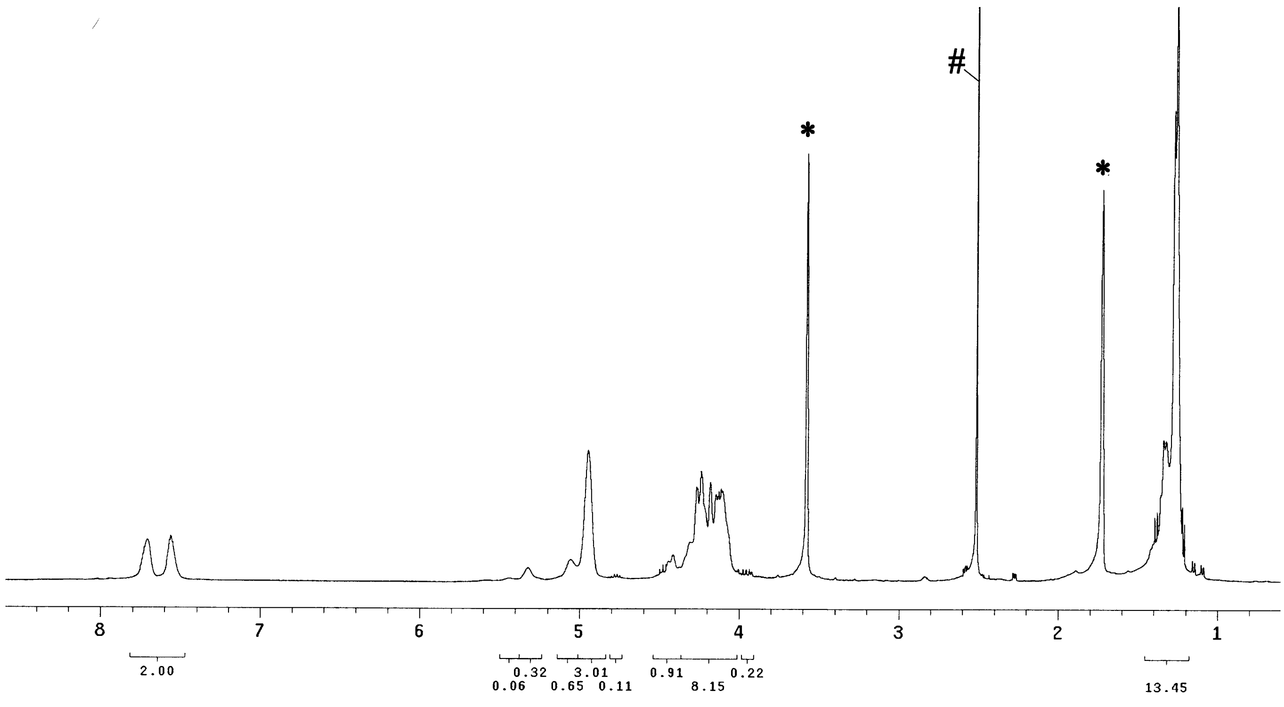

<^{1}H NMR spectrum of CO_2/PO/PA terpolymer prepared using 1.0 g PA and ethanol 15 mg for 4.0 h (entry 15 in Table 2)>

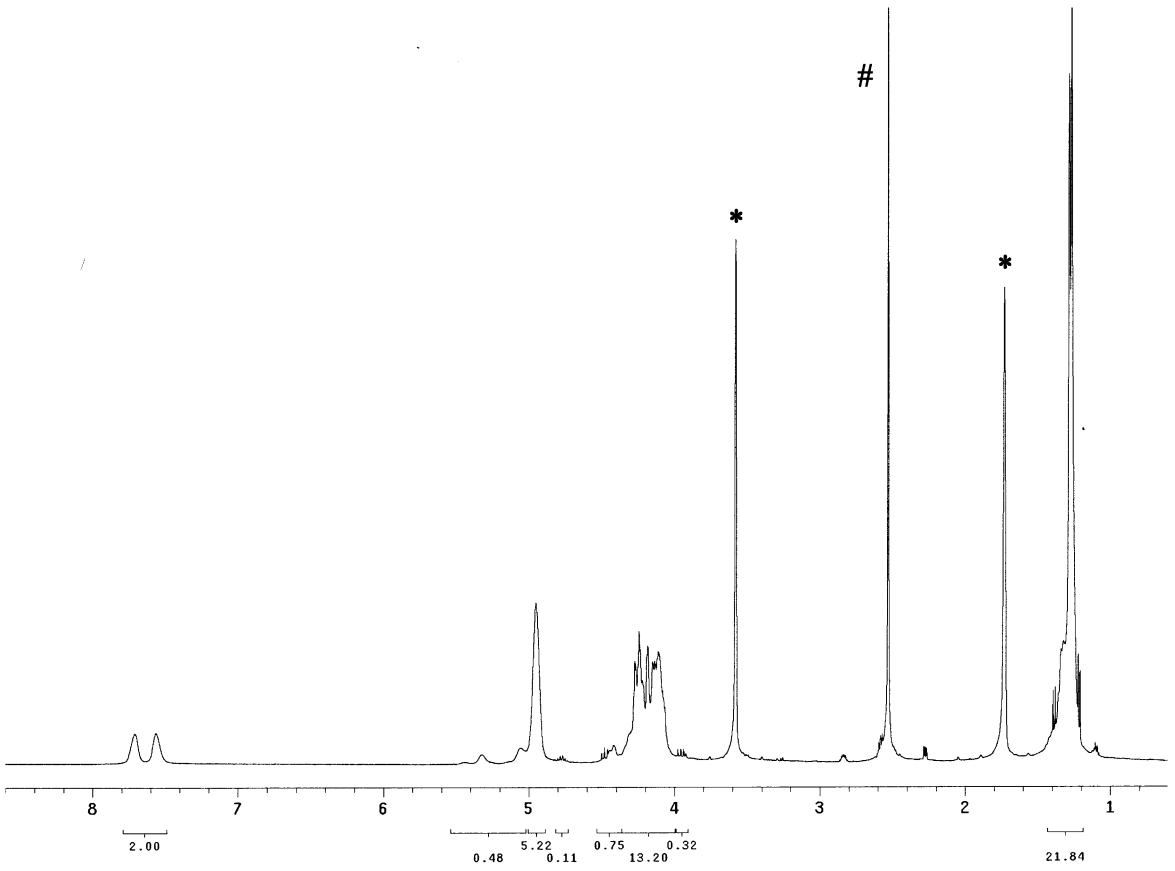

<^{1}H NMR spectrum of CO_2/PO/PA terpolymer prepared using 2.0 g PA and ethanol 15 mg for 3.0 h (entry 16 in Table 2)>

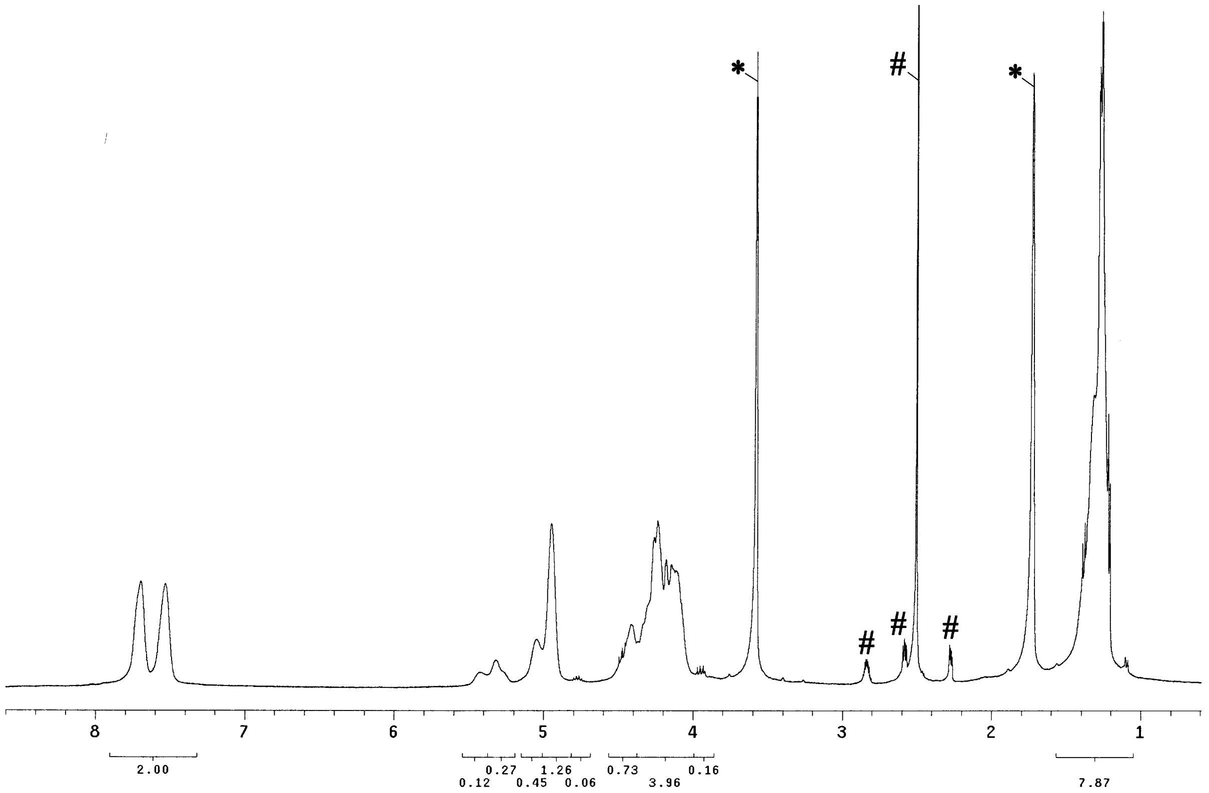

<^{13}C NMR spectrum of PO/PA alternating copolymer prepared using 1.0 g PA for 3.0 h (entry 1 in Table 1)>

The signals marked with "*" is the one of $CDCl_3$.

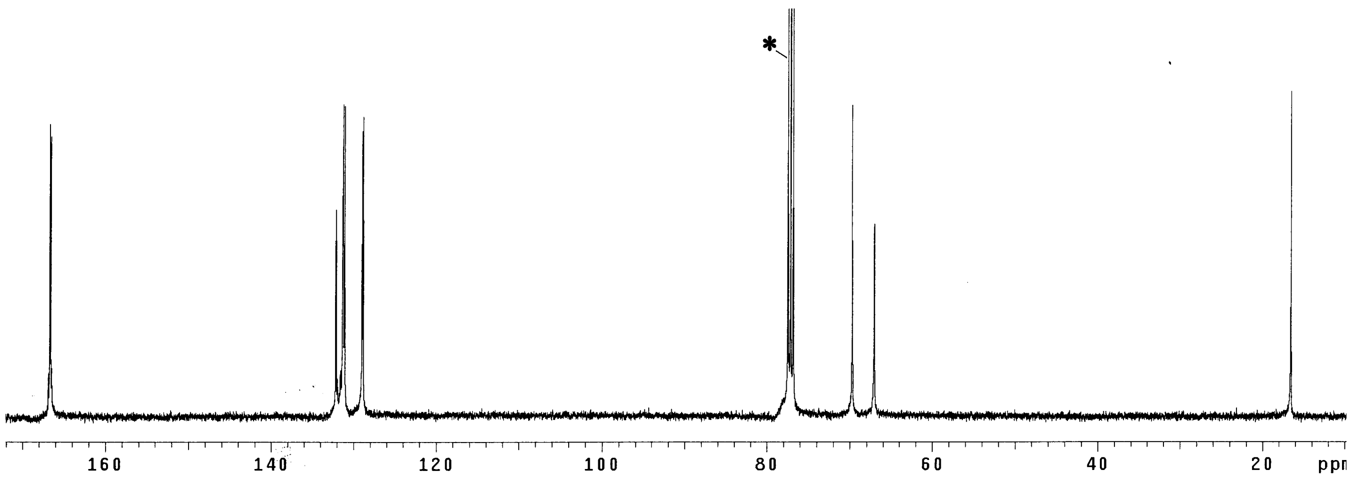

<^{13}C NMR spectrum of CO_2/PO/PA terpolymer prepared using 2.0 g PA for 3.0 h polymerization (entry 7 in Table 2)>

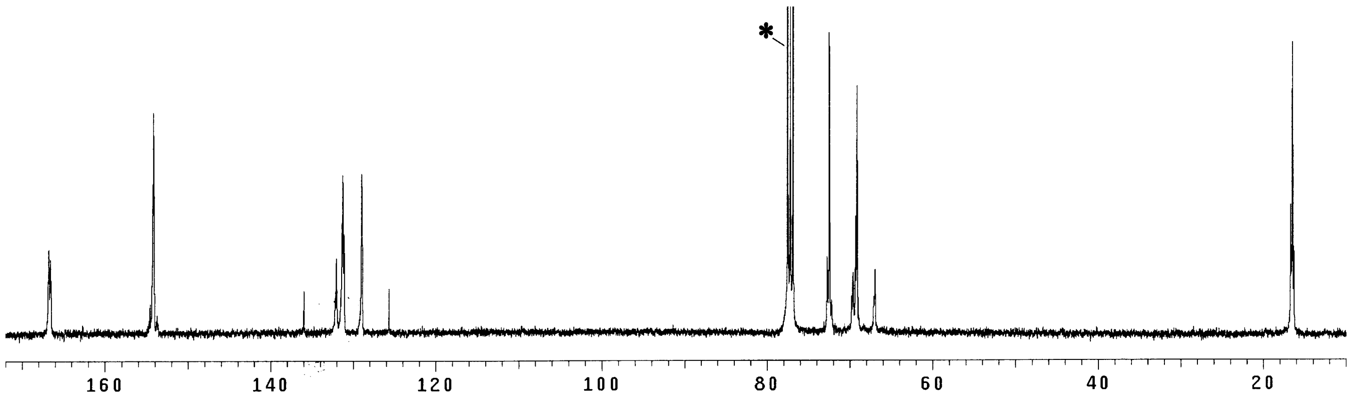

<GPC curves of PO/PA alternating copolymers (entries 2–4 in Table 1)>

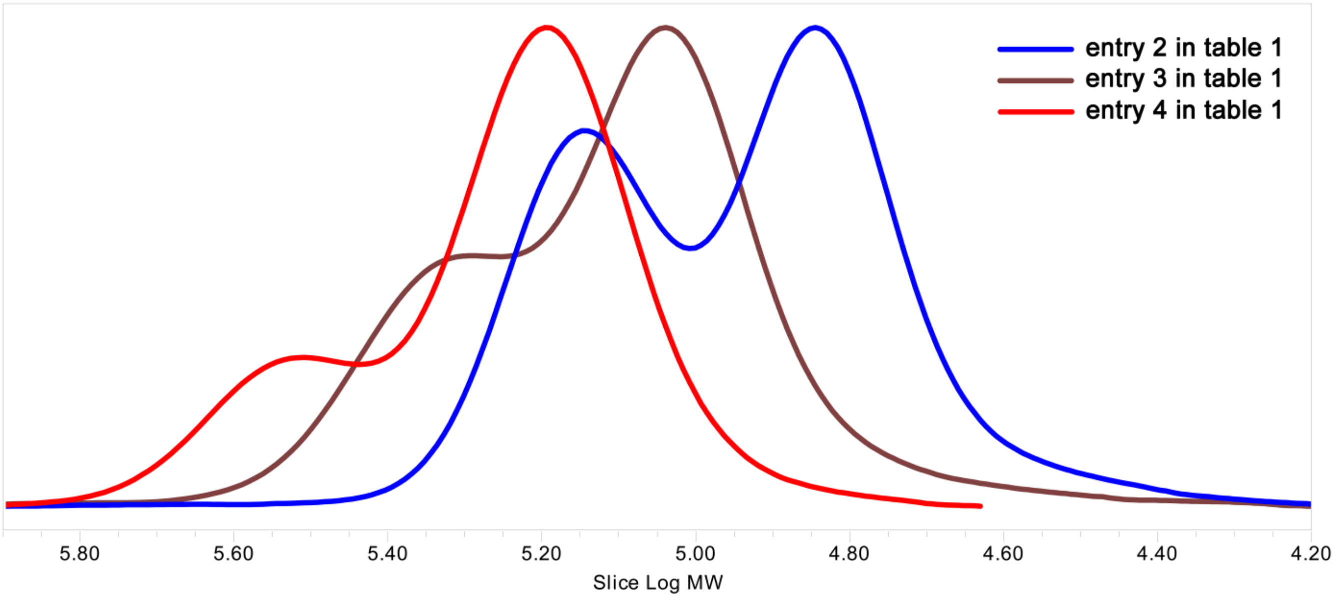

<GPC curves of CO_2/PO/PA terpolymers (entry 1, 2, 4 in Table 2)>

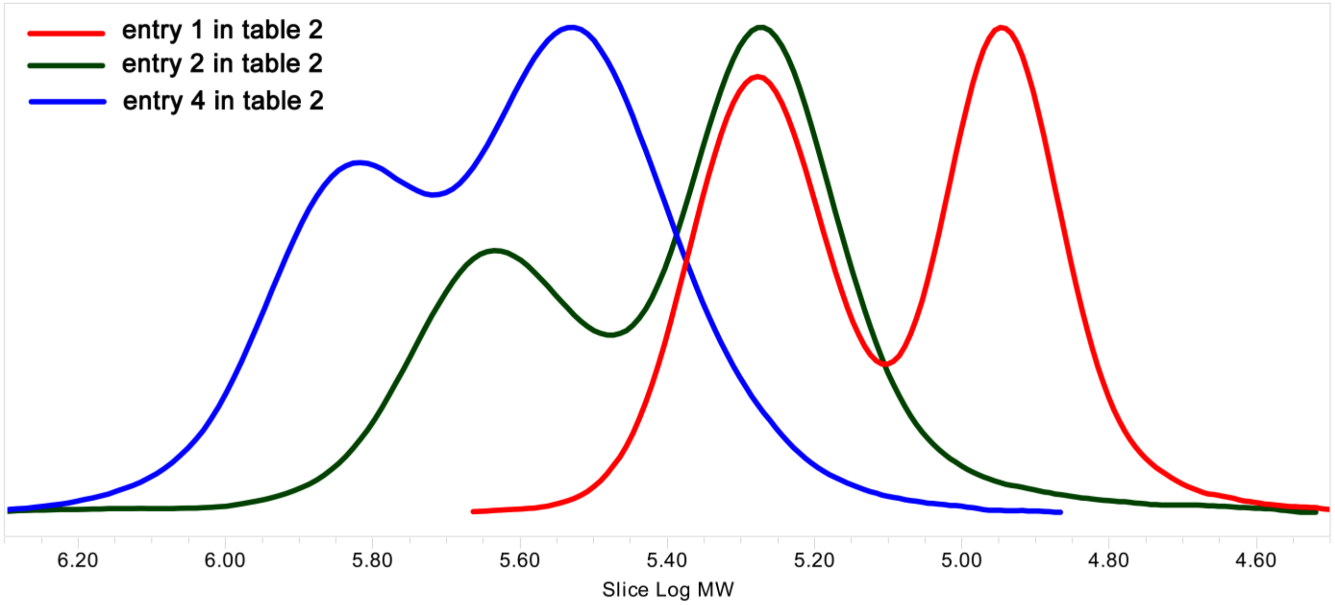

<GPC curves of CO_2/PO/PA terpolymers (entry 5–7, 16 in Table 2)>

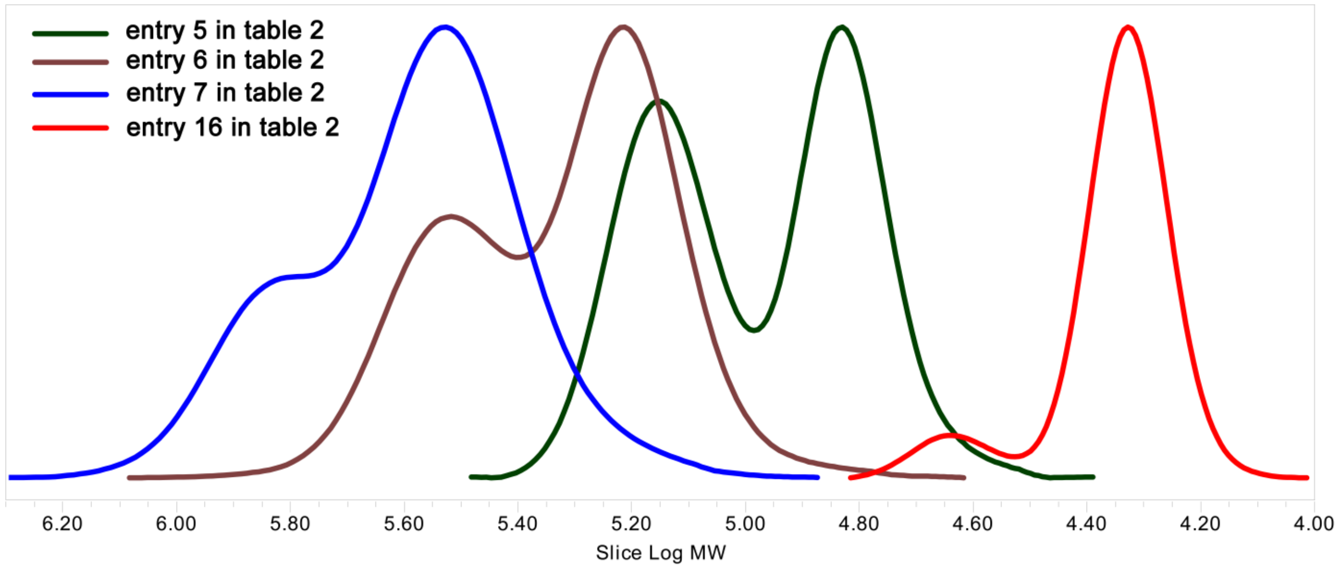

<GPC curves of CO_2/PO/PA terpolymers (entry 1, 8–11 in Table 2)>

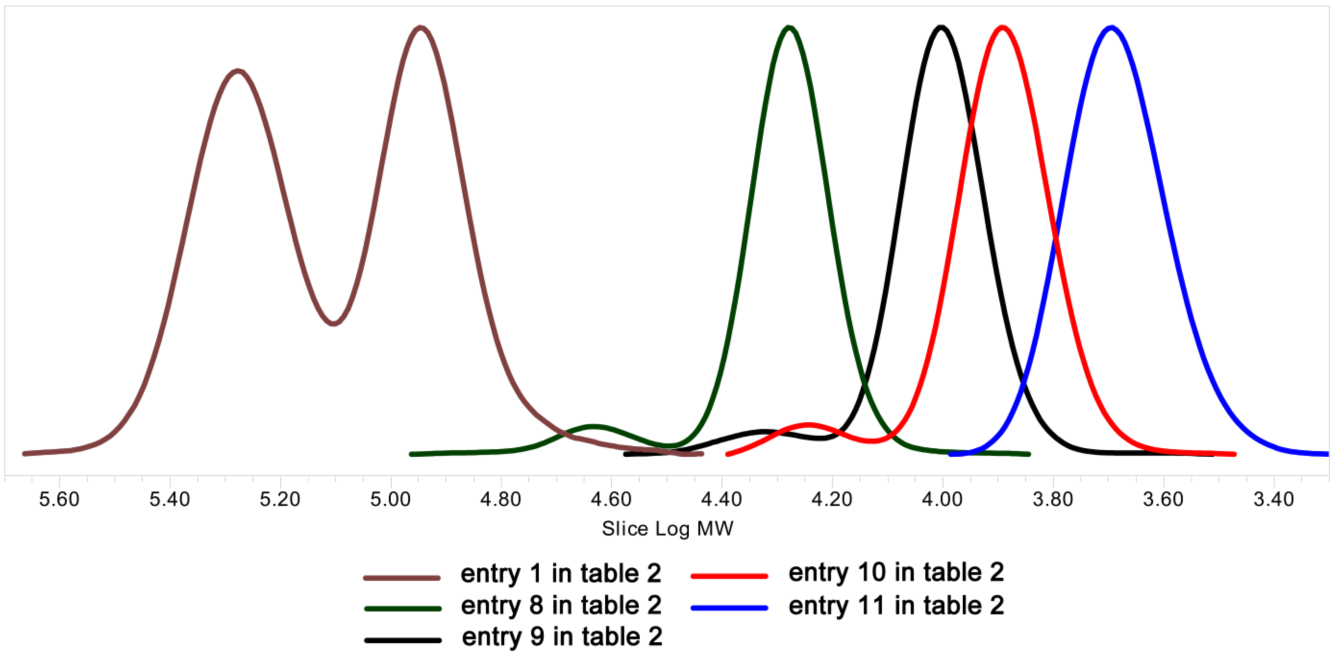

<GPC curves of CO_2/PO/PA terpolymers (entry 10, 12–15 in Table 2)>

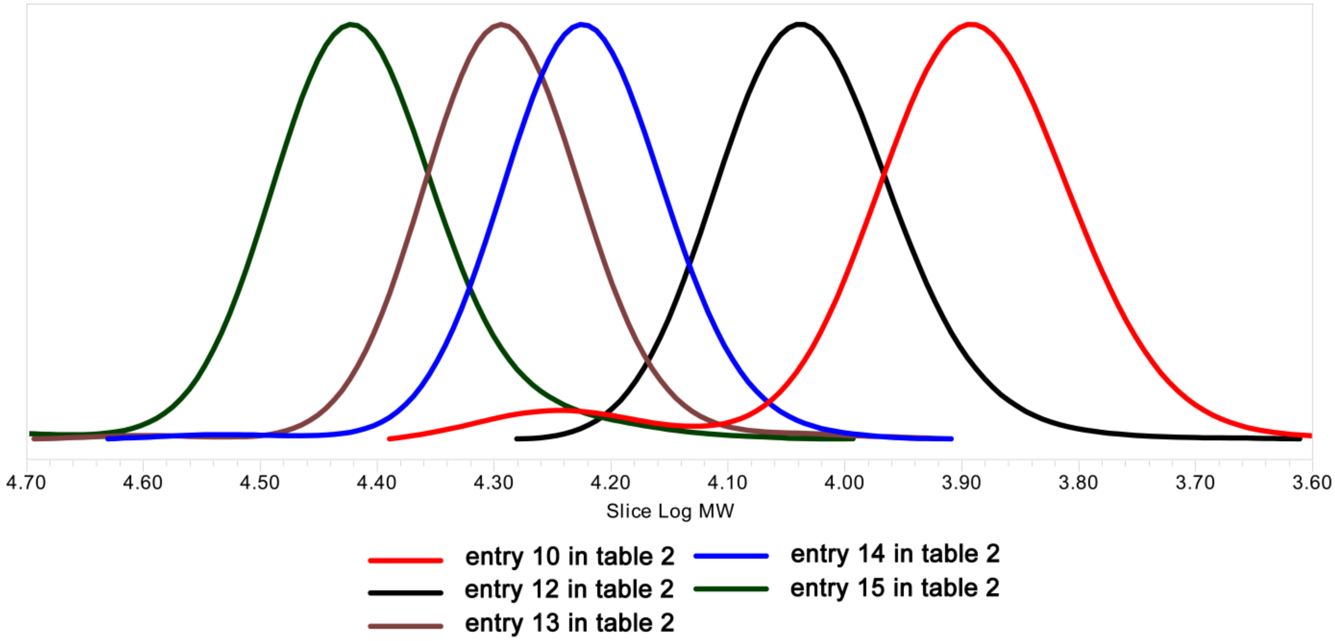

<Picture of the isolated PO/PA alternating copolymer and CO_2/PO/PA terpolymer after removing the catalyst residue>

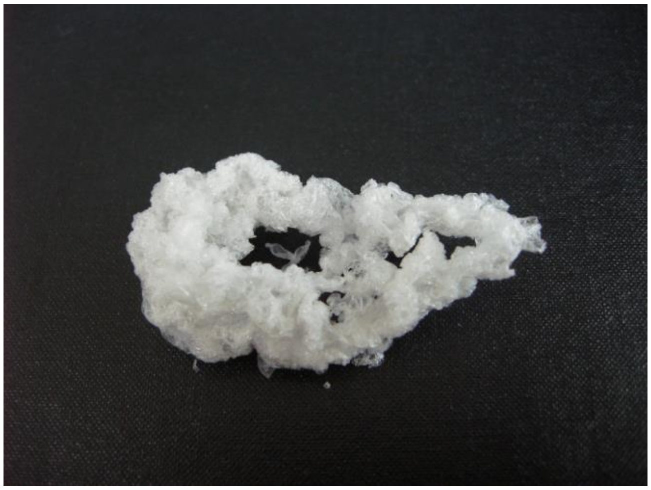

<entry 4 in Table 1>

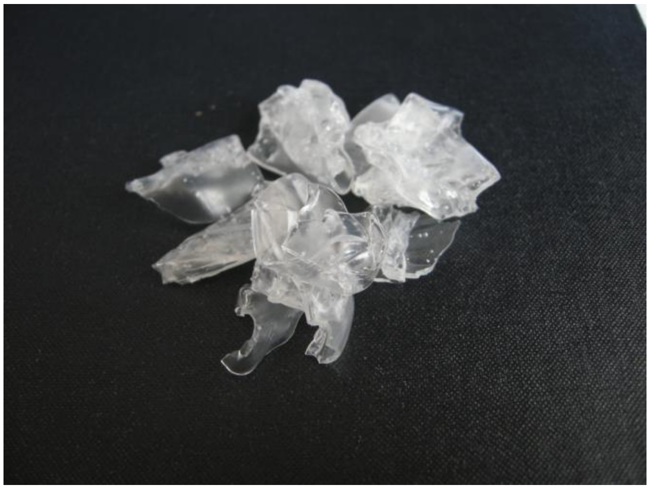

<entry 7 in Table 2>
